# Alzheimer’s disease biological PET staging using plasma p217+tau

**DOI:** 10.1038/s43856-025-00768-z

**Published:** 2025-02-27

**Authors:** Azadeh Feizpour, Vincent Doré, Natasha Krishnadas, Pierrick Bourgeat, James D. Doecke, Ziad S. Saad, Gallen Triana-Baltzer, Simon M. Laws, Rosita Shishegar, Kun Huang, Christopher Fowler, Larry Ward, Colin L. Masters, Jurgen Fripp, Hartmuth C. Kolb, Victor L. Villemagne, Christopher C. Rowe

**Affiliations:** 1 Florey Institute of Neuroscience and Mental Health, Melbourne, VIC Australia; 2 Department of Molecular Imaging & Therapy, Austin Health, Melbourne, VIC Australia; 3 The Australian e-Health Research Centre, CSIRO, Melbourne, VIC Australia; 4 The Australian e-Health Research Centre, CSIRO, Brisbane, QLD Australia; 5 Centre for Precision Health, Edith Cowan University, Perth, WA Australia; 6 Neuroscience Biomarkers, Janssen Research and Development, La Jolla, CA USA; 7 Collaborative Genomics and Translation Group, Edith Cowan University, Perth, WA Australia; 8 Curtin Medical School, Curtin University, Perth, WA Australia; 9 Department of Psychiatry, University of Pittsburgh, Pittsburgh, PA USA; 10 Florey Department of Neuroscience and Mental Health, The University of Melbourne, Melbourne, VIC Australia

**Keywords:** Diagnostic markers, Predictive markers, Prognostic markers, Alzheimer's disease, Alzheimer's disease

## Abstract

**Background:**

Plasma phospho-tau biomarkers, such as p217+tau, excel at identifying Alzheimer’s disease (AD) neuropathology. However, their ability to substitute for tau PET to identify AD biological stage is unclear.

**Methods:**

Participants included 248 cognitively unimpaired (CU) and 227 cognitively impaired (CI) individuals, with Janssen plasma p217+tau Simoa® assay, ^18^F-NAV4694 Aβ-PET (A) and ^18^F-MK6240 tau-PET (T) data. Biological PET stages were defined according to the Revised Criteria for Diagnosis and Staging of Alzheimer’s Disease (2024): Initial (A + T-), Early (A + T_MTL_ + ), Intermediate (A + T_MOD_ + ), and Advanced (A + T_HIGH_ + ). The threshold for A+ was 25 Centiloid and for T_HIGH_ + , the 75th percentile SUVR_temporo-parietal_ in A + CI. Sixty percent were A + , 36% Intermediate/Advanced, and 9% Advanced. The performance of p217+tau in discriminating AD stages was assessed using Receiver Operating Characteristic (ROC) analysis and logistic regression.

**Results:**

Plasma p217+tau concentrations increase across the AD biological PET stages, except between Initial and Early stages. Screening for all AD stages (*vs*. A-T-), combined Intermediate/Advanced stages, or Advanced stage yields AUC of 0.92, 0.92, and 0.91, respectively (CI only: AUC 0.93, 0.89, 0.83). Plasma p217+tau Youden threshold provides sensitivity of 0.77 [0.73–0.90], specificity 0.91 [0.80–0.95], PPV 0.84 [0.71–0.89], and NPV 0.88 [0.85–0.93] for combined Intermediate/Advanced stages. For the Advanced stage alone, sensitivity is 0.89 [0.79–0.97], specificity 0.82 [0.75–0.9], NPV 0.99 [0.98–1.0], but PPV is only 0.33 [0.25–0.47].

**Conclusions:**

In addition to accurately screening for A+ individuals, plasma p217+tau is useful for identifying a combined Intermediate/Advanced stage AD cohort or pre-screening to reduce the tau-PET required to identify Advanced stage AD individuals.

## Plain language summary

Alzheimer’s disease (AD) can be classified into four biological stages: Initial, Early, Intermediate, and Advanced, typically determined using expensive brain scans. We aimed to investigate whether a cost-effective blood test could accurately predict these stages. Our findings show that changes in blood markers can reliably identify individuals in the Intermediate or Advanced stages but are less effective at distinguishing those in the Advanced stage. This suggests that the blood test could help identify individuals with Intermediate to Advanced AD and reduce the need for costly brain scans in some cases.

## Abstract (web summary)

Feizpour et al. assess the utility of a high-performing Alzheimer’s disease (AD) blood test to discriminate AD disease stages. The blood test reliably detects Intermediate and Advanced stages of disease, and while the test is less effective at distinguishing Advanced stage alone, it may reduce reliance on expensive brain scans.

## Introduction

The in vivo staging of Alzheimer’s disease (AD) has become increasingly important, particularly in light of the recent breakthroughs in disease-modifying therapies for AD suggesting less benefit in those with high brain tau^1^. Positron Emission Tomography (PET) imaging allows in vivo staging however, the cost and limited scalability underscores the need to assess plasma biomarkers for this purpose. Among a range of plasma biomarkers, measures of phosphorylated tau (p-tau) at amino acid 217 (p-tau217) have demonstrated considerable potential for detection of AD pathology, rivalling the gold-standards of amyloid-beta (Aβ) PET and cerebrospinal fluid (CSF)^2^.

The plasma p217+tau assay is a high sensitivity Simoa® assay using a capture antibody (pT3), raised against tau paired helical filaments (PHF), that binds to phosphorylation at aa217 (p217) with enhanced binding when other nearby phosphorylated sites are present, predominantly at aa212. It has shown similar performance to other plasma p-tau217 measures in detecting Aβ status in CSF, distinguishing clinical diagnostic groups and predicting progression from Mild Cognitive Impairment (MCI) to AD^3^. A comparison of its performance to recent-generation PET tracers has demonstrated similarly good diagnostic accuracy^4^. Furthermore, we have previously shown that p217+tau predicted cognitive decline and if used as a pre-screening tool in pre-clinical AD trials or screening tool in MCI/AD trials, substantial cost reduction could be achieved^5^. While accumulating evidence underscores the excellent diagnostic, prognostic, and clinical trial utility of plasma p-tau biomarkers, data on their performance for disease staging is sparse^6,7^. Disease staging needs to be assessed at both group level and individual level. Group-level analysis establishes receiver operating characteristic (ROC)-based thresholds (such as 90% sensitivity or 90% positive predictive value (PPV) thresholds) that have proven useful in enriching clinical trial populations. Individual-level analysis provides the foundation for development of risk assessment models aimed at the identification of individuals at elevated risk for specific stages of AD to tailor their prognosis and treatment.

Jack et al.^7^ examined whether the Lilly p-tau217 Meso Scale Discovery (MSD) assay could discriminate between AD disease stages in CU. They defined four tau PET topographic stages using Braak-like PET staging schemes^8,9^. Group-level results indicated good discrimination between those with intermediate (Braak III/IV) or high (Braak V/VI) tau stage vs those with low or no tau. Mattsson-Carlgren et al. ^6^ also used the Lilly p-tau217 MSD assay to stage AD but used those in the top 25% of neocortical PET tau as the high tau group as done in the Lilly Trailblazer-Alz 2 clinical trial^1^. They demonstrated that the assay had only a modest ability to discriminate moderate from high neocortical PET tau.

In the present study, we were interested in both individual-level and group-level discrimination of disease stages. Therefore, we employed the biological PET staging of AD, recently proposed by the Revised Criteria for Diagnosis and Staging of Alzheimer’s Disease (2024)^10^ and aimed to assess the ability of plasma p217+tau using the widely available Simoa® platform to discriminate individuals that fall within these stages. We demonstrate that plasma p217+tau is not only effective for accurately screening amyloid PET positive individuals, but also useful in identifying those at Intermediate or Advanced stages of AD.

## Methods

### Participants

Since 2018, all participants recruited to the AIBL observational study and the allied ADNeT trial screening programme have had ^18^F-MK6240 tau PET, ^18^F-NAV4694 Aβ PET and blood collection at entry. At the time of this study, 506 participants from AIBL and ADNeT who had both Aβ and tau PET scans as well as blood assay results were included. These participants formed a convenience sample for this retrospective study. Details of AIBL recruitment and evaluation are described elsewhere and involved both advertising to the general community and referrals from memory disorder specialists^11^. ADNeT participants were evaluated as per AIBL methods and criteria. Briefly, participants were clinically classified as cognitively unimpaired (CU), mild cognitive impairment (MCI), Alzheimer’s disease (AD) dementia or non-AD dementia, by a multi-disciplinary panel blind to PET imaging and blood assays results. Participants with diagnostic features of other neurodegenerative diseases were excluded. A diagnosis of CU required performance within 1.5 standard deviations (SD) of the published norms for their age group on selected neuropsychological assessments. A diagnosis of MCI or AD dementia followed internationally agreed clinical criteria^12,13^. The MCI and dementia groups were combined into a cognitively impaired (CI) group for most analyses in this study. For identifying sex, study participants were required to self-report.

### Inclusion and ethics

Specific additional IRB approval was not required for this study. The AIBL study, including the follow-up protocol and subsequent amendments and revisions to the protocol, have been approved by the institutional ethical review committees of Austin Health, St. Vincent’s Hospital, Melbourne (SAGE Project ID Number: 2022/PID06188; SVHM Local Ref ID: HREC 028/06), Hollywood Private Hospital and Edith Cowan University. ADNeT study was approved by the institutional ethical review committee at Austin Health, Melbourne (Project Number: HREC/59189/Austin-2019; Austin Health SSA Reference Number: SSA/59189/Austin-2020). All volunteers gave written informed consent, before participating in study assessments, that all of their data including blood samples could be used for research in the field of dementia, and the study was conducted in accordance with the Helsinki Declaration of 1975. No participant was too far progressed at enrolment to be unable to provide informed consent, and all participants consented to follow-up visits. At each follow-up visit, participants were reconsented, allowing them to update their next of kin details. If a participant could no longer provide informed consent at a follow-up visit, the next of kin that was nominated by them at the last visit was allowed to provide consent.

### PET image acquisition and analysis

Aβ PET imaging involved a 20-min acquisition, performed 50 min after injection of 200 MBq of ^18^F-NAV4694 intravenously. Tau PET imaging was conducted on a separate day, with a 20-min acquisition, performed 90 min after intravenous administration of 185 MBq of ^18^F-MK6240. PET analyses were performed by individuals who were blinded to the clinical data or aims of the study. For Aβ PET scans, spatial normalisation was achieved using CapAIBL^14^ and the results were standardised using the Centiloid (CL) scale^15,16^. For tau PET scans, spatial normalisation was performed using the MR-less CapAIBL PCA-based method^17^. Tau PET scans were scaled using the cerebellar cortex as the reference region. ^18^F-MK6240 standardised uptake value ratio (SUVR) was estimated for three in-house composite regions of interest (ROI): (1) mesial temporal (Me) ROI, comprising entorhinal cortex, amygdala, hippocampus, and parahippocampus, (2) temporoparietal (Te) ROI, consisting of inferior temporal, fusiform, supramarginal and angular gyri, posterior cingulate/ precuneus, superior and inferior parietal, and lateral occipital cortex and (3) rest of neocortex (R) including dorsolateral and ventrolateral prefrontal, orbitofrontal, gyrus rectus, superior and middle temporal, and anterior cingulate^18^. A CL threshold of 25 was selected to discriminate Aβ positive (A + ) *vs*. Aβ negative (A-) PET scans. Previously reported thresholds based on the 95th percentile of A- CU were used to discriminate tau positive (T + ) from tau negative (T-) PET scans—1.18 SUVR for Me, 1.24 SUVR for Te and 1.08 SUVR for R^19^. For tau PET uptake in the moderate SUVR range in the Te ROI, we considered values between 1.24 and 2.68. The upper threshold of 2.68 Te SUVR corresponds to 80 CenTauR^20,21^. The Te upper threshold was derived using the method from the Trailblazer-Alz 2 clinical trial^1^ by defining High tau (A + T_HIGH_ + ) as the top quartile of tau PET results in the Aβ + MCI/AD participants within the entire AIBL/ADNeT studies who have undergone ^18^F-MK6240 tau PET imaging (*n* = 348).

### Plasma p217+tau assay

Fasting blood was collected 2.4 ± 10.4 months from the time of Aβ PET scan and 0.1 ± 4.2 months from the tau PET scan. Plasma from K2-EDTA tubes (7.5 mL S-monovette 01.1605.008, Sarstedt), containing prostaglandin E1 (33 ng/mL of whole blood, Sapphire Biosciences), was centrifuged at room temperature for 10 min at 200 *g* to collect platelet-rich plasma. Then, to provide plasma that was snap frozen within 2 h of collection, it was centrifuged at 800 *g* for 10 min, following which it was stored in vapour phase liquid nitrogen prior to shipping on dry ice from Australia to Janssen R&D, La Jolla, CA, USA. The plasma p217+tau assay was completed on a Single Molecule Array (Simoa®) HD-X platform, blinded to all subject data. The samples were measured in duplicate. The technique has been previously described by Triana‐Baltzer et al.^22^. Plasma p217+tau assay results from two laboratories, Janssen R&D (cohort 1; *n* = 397) and Quanterix Corp, MA, USA (cohort 2; *n* = 317), were combined. This study included all participants from cohort 2, and those unique to cohort 1 who were not already part of cohort 2 (final *n* = 506). The correlation between the samples common to both cohorts was 0.84 (Spearman’s correlation). Assay scales measured from both laboratories were comparable and no normalisation or transformation was required. Of the 506 samples included, 30 samples had p217+tau values below the Lower Limit of Quantification (LLOQ: 37 fg/ml). However, all of these samples had a coefficient of variation (CV) of less than 20% and were therefore included in our analyses.

### Implementation of the 2024 criteria for staging of AD

In the Revised Criteria for Diagnosis and Staging of Alzheimer’s Disease (2024)^10^, the following biological PET staging scheme is proposed: 1. Initial stage: proposed as abnormal Aβ with no tau uptake (A + T-), 2. Early stage: as abnormal Aβ with tau uptake limited to medial temporal region (A + T_MTL_ + ), 3. Intermediate stage: as abnormal Aβ with moderate tau uptake in a neocortical ROI (A + T_MOD_ + ), and 4. Advanced stage: as abnormal Aβ with high tau uptake in the same neocortical region (A + T_HIGH_ + ). For stages 3 and 4, we considered Te as our neocortical ROI. Table 1 displays our selected CL and SUVR cut-offs and ranges for defining the aforementioned stages.

**Table 1:** Biological PET staging

PET stages	Centiloid	Tau (SUVR)		
		Me	Te	R
Initial: A + T-	≥25	<1.18	<1.24	<1.08
Early: A + T_MTL_ +	≥25	≥1.18	<1.24	<1.08
Intermediate: A + T_MOD_ +	≥25	<or ≥1.18	Between 1.24 and 2.68	_____
Advanced: A + T_HIGH_ +	≥25	<or ≥1.18	≥2.68	_____
In addition, we considered an A-T- group, to allow comparison:				
**A-T-**	**<25**	**<1.18**	**<1.24**	**<1.08**

*SUVR* standardised uptake value ratio, *Me* mesial temporal ROI, *Te* temporoparietal ROI, *R* rest of neocortex ROI.

Of the 506 participants with plasma p217+tau results, 31 were A- but T + . These were excluded from subsequent analyses as they did not fit the Revised Criteria for Staging AD. Among these, 11 had mildly elevated MTL binding and were postulated to have primary age related tauopathy (PART) but 9 were positive on quantification in MTL and temporo-parietal regions. This proportion of the total study sample of ~2% as having clearly positive neocortical tau PET but negative amyloid PET is consistent with our previous report^23^ (see Supplementary Table 1 for demographic characteristics of the excluded participants).

### Statistics and reproducibility

Data analyses were completed using Python (version 3.9.13), unless otherwise specified. Between-group comparisons for quantitative variables were conducted using one-way analysis of variance (ANOVA) followed by Tukey’s Honest Significant Difference (HSD) where residuals were normally distributed, and the Kruskal-Wallis test followed by Dunn’s test for non-normally distributed residuals. Categorical (Sex, Apolipoprotein E (*APOE*) ε4 allele carriership, clinical stage) comparisons were performed with chi-square (χ²) test. Values are reported as mean ± standard deviation (SD) when normally distributed and median (interquartile range (IQR): Q1-Q3) when skewed. To investigate the magnitude of differences in plasma p217+tau concentration across the PET stages, effect sizes were estimated using Cohen’s *d*, as well as median fold change in concentrations. ROC analysis was used to assess group-level discriminatory capability. The area under the ROC curve (AUC) from each comparison was used to assess the performance of p217+tau alone or in combination with other predictors (age, sex and *APOE* ε4 status) in discriminating between different biological PET stages. The AUCs were compared using DeLong test. Specificity, sensitivity, PPV and negative predictive value (NPV) were reported with bootstrapped 95% confidence intervals (shown in square brackets). PPV and NPV were not adjusted for disease prevalence. Optimal p217+tau thresholds for discriminating between the biological PET stages were derived using Youden’s Index as well as thresholds set at sensitivity and specificity of 90% or 95%. When using a two-threshold approach, participants were classified to Low, Indeterminate and High zones for Aβ PET positivity. Given that there were only nine participants with p217+tau values above 500 fg/ml, with a range from 507 to 902 fg/ml (6 of whom were A + T_HIGH_+ and 3 were A + T_MOD_ + ) and considering the limited statistical power to estimate the line of best fit, we restricted our regression analyses to plasma p217+tau concentrations between 0 and 500 fg/ml in the subsequent sections. To evaluate the probability of a participant belonging to a distinct PET stage (individual patient-level staging), we employed two methods. In the first approach, we computed probability scores by applying a binary logistic regression model. In this model, we used plasma p217+tau as the predictor (independent variable) and grouped PET stages as the outcome (dependent variable), where the stages were categorised into binary groups of 0 and 1. Then, we compared the results of the logistic regression to the second approach, a sliding window method. Here, we calculated PET stage probabilities within sequential bands of plasma p217+tau concentrations, with each band spanning 80 fg/ml. We selected an 80 fg/ml band width as a balance between achieving overly smoothed curves and avoiding excessive noise. The calculated probability for each band was centred at the midpoint of the p217+tau concentration range. The probability estimates from both methods were plotted against p217+tau concentrations and compared across the biological PET stages. All *p* values were two-sided and corrected for multiple comparisons. All analyses were performed across all participants as well as a cognitively impaired only group.

### Reporting summary

Further information on research design is available in the Nature Portfolio Reporting Summary linked to this article.

## Results

### Participant demographics

Demographic characteristics of the participants are presented in Table 2. Participants included 248 CU, 144 with MCI and 83 with dementia (total *n* = 475). Among these, 192 were categorised as A-T-, 80 as A + T-, 31 as A + T_MTL_ + , 128 as A + T_MOD_ + , and 44 as A + T_HIGH_ + . The A + T_HIGH_+ group had a younger average age, compared to the other groups. No significant sex differences were observed among the groups. Relative to A-T-, all other groups displayed higher rates of *APOE* ε4 carriership, higher CDR-SoB score and greater prevalence of MCI or dementia diagnosis. These groups also exhibited higher Centiloid values and plasma p217+tau concentrations. The A + T_MOD_+ and A + T_HIGH_+ had lower MMSE scores. For a breakdown of participant demographics by clinical diagnosis, see Supplementary Table 2.

**Table 2:** Demographic characteristics

	A-T-	A + T-	A + T_MTL_ +	A + T_MOD_ +	A + T_HIGH_ +	*p*
Sample size	192	80	31	128	44	—
Age (years), mean ± SD	74.1 ± 6.2	76.0 ± 7.2	75.2 ± 8.4	75.5 ± 7.1	68.3 ± 7.8***	<0.001
Education (years), median (IQR)	15.0 (11.0–16.0)	11.5 (10.0–16.0)	12.0 (11.0–15.0)	12.0 (11.0–15.0)	11.0 (10.0–15.0)	0.010
Sex, Female (%)	50	42	45	54	50	0.589
APOE ε4 (%)	21	54***	74***	71***	61***	<0.001
MMSE, median (IQR)	29.0 (28.0–30.0)	29.0 (26.0–29.5)	27.0 (25.0–29.0)	26.0 (23.0–28.0)***	24.0 (22.0–25.0)***	<0.001
CDR SoB, median (IQR)	0.0 (0.0–0.5)	0.5 (0.0–1.9)*	0.5 (0.0–3.2)**	1.5 (0.5–4.0)***	4.0 (3.5–5.0)***	<0.001
Centiloid, median (IQR)	−0.7 (−3.8–4.4)	69.5 (43.3–102.4)***	86.7 (59.7–126.7)***	120.9 (90.2–145.7)***	120.5 (101.0–139.9)***	<0.001
MK6240 SUVR_Me_ median (IQR)	0.9 (0.8–1.0)	1.0 (0.8–1.0)	1.3 (1.3–1.5)***	1.8 (1.5–2.1)***	2.2 (1.9–2.7)***	<0.001
MK6240 SUVR_Te_ median (IQR)	1.0 (0.9–1.1)	1.0 (1.0–1.1)	1.1 (1.1–1.2)	1.7 (1.4–2.2)***	3.5 (3.0–4.0)***	<0.001
Plasma p217+tau, fg/ml, median (IQR)	63.5 (46.0–84.9)	118.7 (84.0–156.8)***	140.7 (112.7–168.6)***	211.1 (147.8–288.7)***	336.8 (256.9–425.0)***	<0.001
MCI or Dementia (%)	19	40***	55***	78***	95***	<0.001

*SD* Standard deviation, *IQR* interquartile range, *MMSE* mini-mental state examination, *CDR-SB* clinical dementia rating sum of boxes, *Me* mesial temporal ROI, *Te* temporoparietal ROI, *MCI* Mild Cognitive Impairment, *A-T-* amyloid negative and tau negative, *A* + *T-* amyloid positive and tau negative, *A* + *T*_*MTL*_*+* amyloid positive with tau uptake limited to medial temporal region, *A* + *T*_*MOD*_*+* amyloid positive with moderate tau uptake in temporo-parietal region, *A* + *T*_*HIGH*_*+* amyloid positive with high tau uptake in temporo-parietal region. * p < 0.05, ** *p* < 0.01, *** *p* < 0.001 for comparison to A-T- (Tukey HSD, Dunn’s or chi-square test, with correction for multiple comparisons).

### Plasma p217+tau concentration by PET stages

**Fig. 1:**
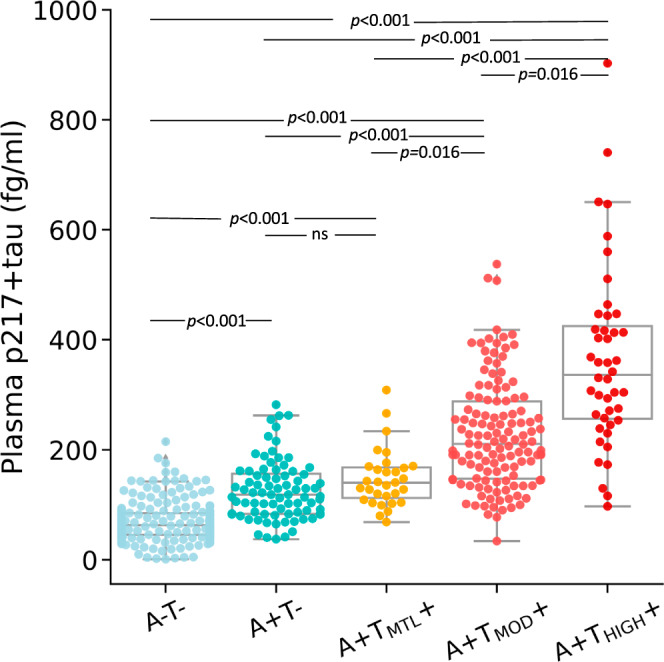
Plasma p217+tau levels in the different biological PET stages. Plasma p217+tau levels in the different biological PET stages. Centre line in each box is the median. The error bars in the boxplots represent 1.5 times the interquartile range (IQR) above and below the upper (75th percentile) and lower (25th percentile) quartiles. The outliers are values that fall below Q1–1.5 × IQR or exceed Q3 + 1.5 × IQR; Q1: the 25th percentile; Q3: the 75th percentile; IQR = Q3–Q1. Plasma p217+tau concentrations exhibited a clear incremental trend across these stages, with significant differences between all, except for the Initial and Early stages. *A-T-* amyloid negative and tau negative (*n* = 192); *A* + *T-* amyloid positive and tau negative (*n* = 80); *A* + *T*_*MTL*_*+* amyloid positive with tau uptake limited to medial temporal region (*n* = 31); *A* + *T*_*MOD*_*+* amyloid positive with moderate tau uptake in temporo-parietal region (*n* = 128); *A* + *T*_*HIGH*_*+* amyloid positive with high tau uptake in temporo-parietal region (*n* = 44). *ns* non-significant. Pairwise comparison using Dunn’s test, with correction for multiple comparisons.

The median (IQR) concentration of plasma p217+tau exhibited a clear incremental trend across the stages, with values of 63.5 (46.0–84.9) fg/ml in the A-T-, 118.7 (84.0–156.8) fg/ml in A + T-, 140.7 (112.7–168.6) fg/ml in A + T_MTL_ + , 211.1 (147.8–288.7) fg/ml in A + T_MOD_+ and 336.8 (256.9–425.0) fg/ml in A + T_HIGH_ + (Fig. 1). A pairwise comparison—corrected for multiple comparisons— revealed significant differences in mean plasma p217+tau concentration between all stages, except for the comparison between A + T- and A + T_MTL_ + . Henceforth, in all subsequent analyses, these two stages were consolidated into a unified stage referred to as Initial/Early (A+T_None/MTL_). For detailed information on Cohen’s *d* effect sizes, median fold changes and *p*-values for comparison between the stages, see Supplementary Table 3.

### Group-level approach for disease staging

#### Discrimination between A-T- and A+ stages

**Fig. 2:**
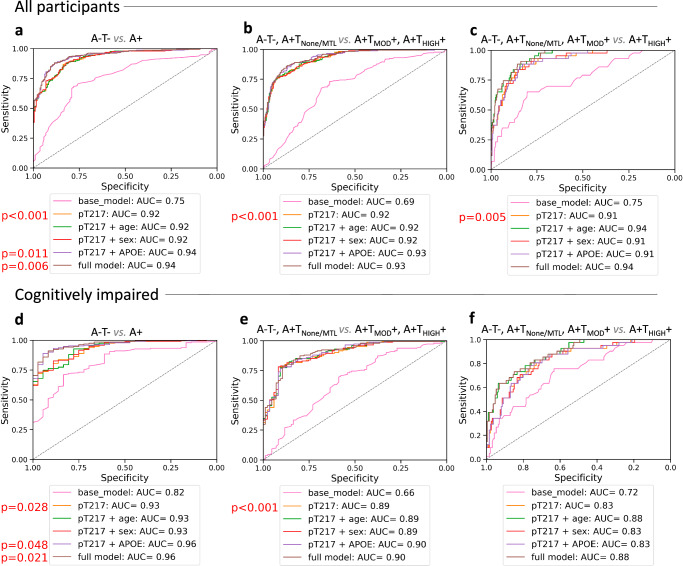
ROC analyses for biological PET staging. ROC analysis for (**a**–**c**) all participants (*n* = 472), and (**d**–**f**) the cognitively impaired group (*n* = 226). Base model includes age, sex, and Apolipoprotein E (*APOE*) ε4 status. Full model includes p217+tau, age, sex, and *APOE* ε4 status. *pT217* is plasma p217+tau; *AUC* area under the receiver operating characteristic curve. *A-T-* amyloid negative and tau negative; *A+T*_*None/MTL*_ amyloid positive and tau negative or amyloid positive with tau uptake limited to medial temporal region; *A* + *T*_*MOD*_*+* amyloid positive with moderate tau uptake in temporo-parietal region; *A* + *T*_*HIGH*_*+* amyloid positive with high tau uptake in temporo-parietal region. Significant *p* values from DeLong test are presented for comparison of the model with p217+tau only, to the base model, along with for comparison of all models (except the base model) to the model with p217+tau only (corrected for multiple comparisons).

When CU and CI participants were combined, plasma p217+tau alone discriminated between A-T- and all stages combined of A + , with an AUC of 0.92 (Fig. 2a), significantly better than the discrimination by the base model which included age, sex and *APOE* ε4 status (AUC: 0.75, *p* < 0.001). The Youden index provided a threshold concentration of 99.4 [89.7–102.4] fg/ml, which yielded sensitivity of 0.87 [0.83–0.91], specificity of 0.84 [0.78–0.88], PPV of 0.89 [0.85–0.92] and NPV of 0.81 [0.77–0.87] (Supplementary Table 4). Adding age or sex separately did not improve the AUC of the model with p217+tau while *APOE* ε4 status improved the model slightly (AUC: 0.94, *p* = 0.011). There was no further improvement using the full model including p217+tau, age, sex and *APOE* ε4 status.

Including the excluded 31 A-T+ individuals in the A- group (total *n* = 506, 56% A + ), minimally affected the AUC 0.91 [0.89–0.93] vs 0.92 [0.90–0.94] and the Youden derived p217+tau threshold was unchanged producing an accuracy of 84%. Applying a two-threshold approach using 95% sensitivity and 90% specificity thresholds correctly classified 91% of those in the high or low range (PPV 90%, NPV 91%) after excluding the 26% in the indeterminate range between the thresholds.

Among CI only participants, plasma p217+tau discriminated between A-T- and A+ individuals with an AUC of 0.93 [0.89–0.96] (Fig. 2d), significantly better than the discrimination by the base model (AUC: 0.82, *p* = 0.028). Youden threshold of 126.7 fg/ml [94.8–149.9 fg/ml] gave a sensitivity of 0.83 [0.69–0.95], specificity of 0.86 [0.79–1.0], PPV of 0.97 [0.96–1.0], and NPV of 0.48 [0.34–0.74] noting that A+ prevalence was 84% in the CI group (Supplementary Table 5). Adding age or sex separately did not improve the AUC of the model with p217+tau while adding *APOE* ε4 status did (AUC: 0.96, *p* = 0.048). There was no further improvement using the full model.

#### Discrimination between combined A-T-/A+T_None/MTL_ and combined A + T_MOD_ + /A + T_HIGH_ + 

In a clinical trial assessing the impact of a disease-modifying treatment on individuals with Aβ and neocortical tau pathology, screening for these individuals *vs*. A-T-/A+T_None/MTL_ individuals may be useful.

Among the CU & CI combined, for discriminating these two groups, using p217+tau alone, the AUC was 0.92 [0.90–0.94] (Fig. 2b), performing significantly better than the base model (AUC: 0.69, *p* < 0.001).Youden index gave a threshold of 168.0 fg/ml [131.8–177.4 fg/ml] which yielded a sensitivity of 0.77 [0.73–0.90], specificity of 0.91 [0.80–0.95], PPV of 0.84 [0.71–0.89], and NPV of 0.88 [0.85–0.93] noting the prevalence of A + T_MOD_ + /A + T_HIGH_+ was 36% (Supplementary Table 4). Adding age, sex or *APOE* ε4 status separately or together did not improve the AUC of the model with p217+tau. Adding MMSE score to the full model also did not assist AD staging (see Supplementary Fig. 1).

Among the CI only, the AUC was 0.89 [0.86–0.93] (Fig. 2e), significantly better than the discrimination by the base model (AUC: 0.66, *p* < 0.001), Youden threshold was 177.4 fg/ml [168.0–179.4 fg/ml], sensitivity 0.78 [0.74–0.86], specificity 0.92 [0.85–0.96], PPV 0.94 [0.89–0.97], NPV 0.72 [0.66–0.81] (Supplementary Table 5). Adding age, sex or *APOE* ε4 status separately or together did not improve the model with p217+tau.

#### Discrimination between A + T_HIGH_+ and combined A-T-/A+T_None/MTL_/A + T_MOD_ + 

Among the CU & CI combined, the use of plasma p217+tau alone for discriminating between these two groups gave an AUC of 0.91 [0.88–0.95] (Fig. 2c), significantly better than the performance of the base model (AUC: 0.75, *p* = 0.005). Adding age, sex or *APOE* ε4 status separately or together did not improve the model with p217+tau. Adding MMSE score to the full model did not assist AD staging (see Supplementary Fig. 1).

Youden index yielded a threshold of 205.4 fg/ml [173.2–257.9 fg/ml] with a sensitivity of 0.89 [0.79–0.97], specificity of 0.82 [0.75–0.90], PPV of 0.33 [0.25–0.47], NPV of 0.99 [0.98–1.0] noting the prevalence of 9% for A + T_HIGH_ + . (Supplementary Table 4).

When only CI participants were included, plasma p217+tau alone discriminated between these two groups with an AUC of 0.83 [0.78–0.89] (Fig. 2f) and this AUC did not significantly differ from any of the other models. Age increased the AUC to 0.88 likely reflecting the younger age of individuals with A + T_HIGH_+ but this increase did not reach significance.

In CI the Youden index provided a threshold of 230.3 fg/ml [205.4–299.5 fg/ml] giving sensitivity of 0.84 [0.69–0.95], and specificity 0.71 [0.64–0.88] (Supplementary Table 5). With PPV of 0.39 [0.31–0.58] and NPV of 0.95 [0.92–0.98], p217+tau effectively excluded Advanced stage AD when below the Youden threshold, but did not accurately predict Advanced stage when above, due to the inclusion of many Intermediate stage AD participants.

For a pairwise comparison of each two biological PET stages across the entire cohort, see Supplementary Table 6.

#### Two step discrimination between Intermediate and Advanced disease stages

Given the suboptimal performance of the Youden index for discriminating those with Advanced stage AD, particularly the PPV of 0.39, we evaluated a two-step Youden approach. After employing the Youden threshold of 177.4 fg/ml for CI participants to identify those who were either A + T_MOD_+ or A + T_HIGH_ + , (total *n* = 117, A + T_HIGH_ + = 37), for discriminating the A + T_HIGH_+ in this selected cohort, the AUC was 0.76 [0.68–0.83], Youden index threshold 299.5 fg/ml [257.9–402.9], sensitivity 0.71 [0.47–0.91], specificity 0.70 [0.52–0.95], PPV 0.52 [0.41–0.79], NPV 0.84 [0.76–0.93].

We also evaluated thresholds chosen for 90% sensitivity, 90% specificity and 95% specificity for A + T_HIGH_ + (see Supplementary Fig. 2) but no threshold was able to provide both satisfactory sensitivity and PPV (e.g., in CI a 90% sensitivity threshold gave PPV of only 32%; a 95% specificity threshold gave PPV of 70% with only 33% sensitivity). Thus, adjusting the threshold to improve the PPV resulted in substantially reduced sensitivity due to the high degree of overlap with Intermediate stage AD, as can be seen in Fig. 1.

### Individual-level approach for disease staging

**Fig. 3:**
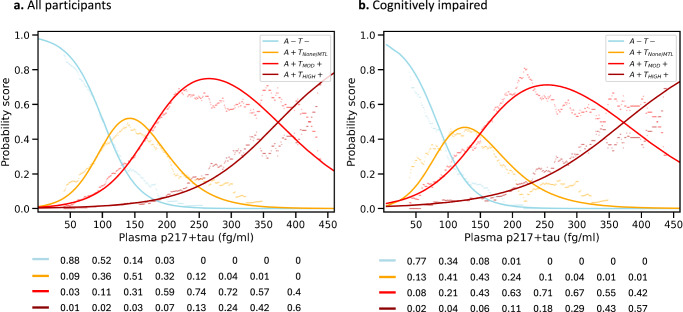
Probability scores for each biological PET stage *vs*. plasma p217+tau concentration. Logistic regression probabilities for (**a**) all participants (*n* = 465; p217+tau values > 500 excluded), and (**b**) the cognitively impaired group (*n* = 218; p217+tau values > 500 excluded). Solid line is the probability scores generated by logistic regression and dots are the probability scores generated by sliding window methods. The tables below the figures outline the probability scores for the different sequential p217+tau levels. *A-T-* amyloid negative and tau negative; *A+T*_*None/MTL*_ amyloid positive and tau negative or amyloid positive with tau uptake limited to medial temporal region; *A* + *T*_*MOD*_*+* amyloid positive with moderate tau uptake in temporo-parietal region; *A* + *T*_*HIGH*_*+* amyloid positive with high tau uptake in temporo-parietal region.

We also performed logistic regressions to ascertain whether an individual’s plasma p217+tau level can inform their classification into A-T-, Initial-Early (A+T_None/MTL_), Intermediate (A + T_MOD_ + ), or Advanced (A + T_HIGH_ + ) stages. Figure 3a (CU & CI combined) and Fig. 3b (CI only) display the logistic regression probability scores for each stage as a function of plasma p217+tau concentrations. At any given plasma p217+tau value, an individual is most likely to belong to the stage with the highest probability score. Individuals with low p217+tau concentrations (below 110 fg/ml for CU & CI combined, or 93 fg/ml for CI only) had the highest likelihood of being A-T-. A+T_None/MTL_ was the classification with the highest likelihood in p217+tau value range of 110–172 fg/ml for CU & CI combined, and 93–150 fg/ml for CI only. A + T_MOD_+ was the most likely classification for a wide range of moderate p217+tau values (172–375 fg/ml for CU & CI combined, and 150–374 fg/ml for CI only). For p217+tau values higher than ~375 fg/ml, the classification of A + T_HIGH_+ was the most likely.

In Fig. 3, the likelihoods from logistic regression (solid line) are compared to the ones estimated using the sliding window method (dots), indicating good concordance between the two approaches in computing probabilities.

## Discussion

In the present study, we evaluated the ability of the Janssen plasma p217+tau Simoa® assay to discriminate between AD disease stages, employing the biological PET stages recommended by the Revised Criteria for Diagnosis and Staging of Alzheimer’s Disease (2024)^10^. With high cost and limited availability of PET, the ability to predict the level and spatiotemporal patterns of AD using plasma biomarkers would improve access for patients and reduce clinical trial cost.

At this time there is no generally accepted specific threshold for the level of neocortical tau required to distinguish the Intermediate from the Advanced stage of AD in the Revised Criteria. We chose the top quartile approach employed in the TRAILBLAZER-ALZ 2 Phase III Donanemab trial^1^ that showed reduced benefit from this amyloid monoclonal antibody when this level of tau was present. This gave a threshold for ^18^F-MK6240 tau PET of 2.68 SUVR in the lateral temporal and parietal cortex and we calculated that this corresponds to 80 CTR in the recently proposed CenTauR method for standardisation of tau PET quantitation^20^.

We found that as disease progressed from A-T- to the Advanced biological PET stage (A + T_HIGH_ + ), there was a clear incremental trend across the stages in plasma p217+tau concentration, reaching more than a five-fold increase. We showed that plasma p217+tau alone outperformed a combination of established risk factors (age, sex, APOE e4) in discriminating biological PET stages. In cognitively impaired participants, p217+tau performed well for discriminating a combined Intermediate/Advanced (i.e., A + T_MOD_+ or A + T_HIGH_ + ) cohort from lower stages with an AUC of 0.89 and the Youden threshold providing 78% sensitivity and 94% PPV. However, plasma p217+tau was not able to accurately isolate the Advanced (A + T_HIGH_ + ) AD stage participants due to substantial overlap with Intermediate stage AD participants. The Youden threshold gave excellent NPV (95%), good sensitivity (84%) but low PPV (39%). Likewise, a double threshold approach and predefined 90% sensitivity thresholds produced high NPV but low PPV.

The pairwise discrimination of each pair of AD biological PET stages — Initial vs. Early, Early vs. Intermediate, and Intermediate *vs*. Advanced — yielded lower AUC values than grouped comparisons. The AUC of 0.64 for Initial *vs*. Early, suggested that p217+tau did not have adequate discriminatory performance for these two stages. The AUC of 0.76, observed in the discrimination between Early and Intermediate stages, falls within an acceptable yet relatively lower range. This highlights that the higher AUC of 0.92 reported for the A-T-/Initial/Early *vs*. Intermediate/Advanced discrimination is strongly influenced by the inclusion of all lower stages. The same reasoning applies to the AUC of 0.77 for Intermediate *vs*. Advanced compared to the AUC of 0.91 for Advanced *vs*. all others.

The overlap between the range of plasma p217+tau levels was again apparent in a risk prediction model using p217+tau as the predictor and estimated probability scores for an individuals’ absolute risk of falling within a disease stage. With the overlap in likelihood functions for most PET stages, confident staging of an individual with plasma p217+tau alone is limited to very low or very high levels (Fig. 3).

Our study findings are consistent with recent reports from two other groups. Mattsson-Carlgren et al.^6^ used the Lilly pTau217 MSD assay to identify those with top quartile tau PET levels using the tau PET tracer ^18^F-RO948. Although the whole cohort AUC was 0.90, in CI it showed more modest performance at 0.84 (our AUC 0.91 and 0.83 respectively), likewise due to substantial overlap with moderate tau subjects. Using a threshold set for a 10% false negative rate as a rule out pre PET screen, they calculated a potential 50% reduction in the number of tau PET required to establish a high tau PET cohort. At a threshold set a 90% sensitivity, we obtained 96% NPV in CI and 32% PPV providing a cohort enriched three fold for the Advanced AD stage suggesting a similar potential tau PET cost saving when creating a high tau cohort. Jack et al.^7^ who also used the Lilly p-tau217 MSD assay but only in CU and Quanterix Simoa p-tau181 in CU and CI used Braak stages from ^18^F-flortaucipir tau PET to define AD stage and reported good group-level discrimination with plasma p-tau level. However, 77% of the CI in Jack’s study were in the high tau stage defined as Braak stage V/VI limiting comparisons to our study.

Previous studies have shown that the tau tracer ^18^F-MK6240 might be better suited to detect early tau accumulation (Me ROI) compared to ^18^F-Flortaucipir^24,25^. Despite this we were not able to show that plasma p217+tau was able to discriminate A + T- from A + T_MTL_ + .

Consequently, the value of plasma p217+tau appears to lie in the detection of A+ individuals and to reduce the required number of tau PET for trial recruitment by defining a combined Intermediate/Advanced AD stage cohort where tau PET can then be used to separate Advanced from Intermediate stage. Similarly with a 95% NPV for Advanced stage AD when applied across all CI participants, plasma p217+tau may be used to screen out persons unlikely to have Advanced AD, with tau PET then required to separate Advanced AD from lower stages in those above the plasma p217+tau threshold.

Further development of risk prediction models using a combination of biomarkers to more accurately predict individual-level disease stages is required. In a recent review by Therriault J et al.,^26^ it was postulated that more accurate staging of AD might be achieved by a combination of fluid biomarkers that sequentially become abnormal as AD disease stage progresses. Changes in a variety of CSF biomarkers reflect progression of AD pathology starting with reductions in the Aβ42/40 ratio, followed by increases in p-tau217, p-tau205, MTBR-tau243 and finally, non-phosphorylated mid-region tau^27^. CSF MTBR-tau243 measured by mass spectrometry is a specific marker of tau tangle pathology in AD^27^ and preliminary data suggests that measurement in plasma may yield similar findings^28^.

A limitation of our study is that the findings are derived from a research cohort with a relatively high prevalence of Aβ-PET positivity in the three clinical groups. Replication of prevalence-dependent findings (i.e., PPV, NPV, estimated probability scores, cut-off values) in a real-world memory clinic setting is needed before any of the findings could be applied in clinical practice. With some coefficients of variance for plasma p217+tau samples nearing 20%, this variability should be taken into account when considering the assay for diagnostic use.

To conclude, plasma p217+tau performs well for detecting persons with brain amyloid and discriminating those with Intermediate or Advanced stages from those at a lower stage, a useful property to reduce the number of tau PET required when screening for individuals at a particular stage of AD.

## Supplementary information

Transparent Peer Review file

Supplementary Information

Description of Additional Supplementary Files

Supplementary_Data_1 to 3

Reporting Summary

## Data Availability

The anonymized ADNeT data reported in the manuscript are available from the corresponding author upon reasonable request, from a qualified academic investigator for the purpose of replicating the results presented in the article. Access to the deidentified AIBL data can be achieved by submitting an expression of interest (EOI) to AIBL via www.aibl.csiro.au. The source data for Figs. 1–3 is in Supplementary Data 1–3.

## Code availability

The analyses code used to produce the results presented in this paper is available at: 10.5281/zenodo.14768617^29^.
