# Supplementary material for: Alzheimer’s disease biological PET staging using plasma p217+tau
Source: Commun Med (Lond). 2025 Feb 27;5:53. doi: 10.1038/s43856-025-00768-z (PMC11868538; doi:10.1038/s43856-025-00768-z)
Supplement: Supplementary file 2 — Supplementary Information [file 43856_2025_768_MOESM2_ESM.pdf]

Supplementary Information

Supplementary Table 1. Demographic characteristics of participants that did not fit the NIA-AA based profiles or the A-T- group

	A-T$_{Me+Te-R-}$	A-T$_{Me-Te+R-}$	A-T$_{Me-Te-R+}$	A-T$_{Me-Te+R+}$	A-T$_{Me+Te+R-}$	A-T$_{Me+Te+R+}$
Sample size	11	2	6	3	4	5
Age (years), mean ± SD	76.4 ± 5.8	79.0 ± 3.0	74.7 ± 5.2	67.0 ± 5.0	78.0 ± 4.1	70.2 ± 4.3
Education (years), median (IQR)	15.0 (11.5-15.5)	12.0 (10.5-13.5)	16.0 (13.0-16.8)	15.0 (13.0-18.5)	15.0 (14.0-15.0)	12.0 (9.0-12.0)
Sex, Female (%)	45	50	83	67	75	40
MMSE, median (IQR)	27.0 (24.5-30.0)	29.5 (29.2-29.8)	29.0 (29.0-29.8)	27.0 (26.0-28.5)	26.5 (24.5-27.5)	23.0 (22.0-24.0)
CDR SoB, median (IQR)	0.5 (0.0-2.0)	0.2 (0.1-0.4)	0.0 (0.0-0.0)	1.5 (0.8-3.8)	1.0 (0.5-1.9)	3.5 (1.5-4.5)
MK6240 SUVR$_{MetaT}$, median (IQR)	1.2 (1.1-1.3)	1.1 (1.1-1.2)	1.0 (0.9-1.0)	1.1 (1.0-1.1)	1.2 (1.2-1.3)	1.2 (1.1-2.0)
Centiloid, median (IQR)	8.0 (-1.3 - 11.5)	-6.2 (-6.9 - -5.6)	-4.3 (-8.8 - -0.8)	-5.3 (-5.5 - -1.8)	-5.2 (-6.6 - 0.7)	9.7 (0.9 - 16.6)
Plasma p217+tau, fg/ml, median (IQR)	81.8 (61.5-100.0)	126.2 (97.2-155.1)	49.4 (42.1-60.0)	74.7 (48.3-83.6)	123.3 (107.6-128.5)	101.6 (95.5-212.7)
MCI or Dementia (%)	45	0	17	67	75	80

Me mesial temporal ROI; *Te* temporoparietal ROI; *R* rest of neocortex. Positive sign denotes being above the pre-defined thresholds and negative below the pre-defined thresholds outlined in the Methods section. *SD* Standard deviation; *IQR* interquartile range; *MMSE* mini-mental state examination; *CDR-SoB* clinical dementia rating sum of boxes; *MK6240 SUVR$_{MetaT}$* Tau ^{18}F-MK6240 SUVR estimated in a standard meta-temporal composite region, comprising entorhinal cortex, parahippocampus, amygdala, inferior temporal, fusiform, and middle temporal cortex ROI; *MCI* Mild Cognitive Impairment.

Supplementary Table 2. Demographic characteristics, breakdown by clinical diagnosis

		CU	MCI	Dementia
Sample size		248	144	83
Age (years), mean ± SD		75.6 ± 6.2	73.8 ± 7.7	71.5 ± 8.2
Education (years), median (IQR)		15.0 (11.0-16.0)	11.0 (10.0-15.0)	11.0 (10.0-15.0)
Sex, Female (%)		54	45	43
APOE ε4 (%)		35	62	59
MMSE, median (IQR)		29.0 (28.0-30.0)	27.0 (25.0-28.0)	23.0 (21.0-25.0)
CDR SoB, median (IQR)		0.0 (0.0-0.0)	1.0 (0.5-1.5)	4.5 (4.0-6.0)
MK6240 $SUVR_{MetaT}$, median (IQR)		1.0 (0.9-1.1)	1.5 (1.1-2.1)	2.1 (1.3-2.6)
Centiloid, median (IQR)		5.5 (-1.8-59.3)	101.9 (44.2-129.2)	109.0 (74.3-139.4)
Aβ PET positive (%)		37	81	90
Plasma p217+tau, fg/ml, median (IQR)		81.5 (52.0-126.9)	173.3 (115.5-259.4)	215.2 (127.8-350.5)
AT biomarker groups	A-T- (%)	63	19	10
	A+T- (%)	19	16	11
	A+T_{MTL}+ (%)	6	7	8
	A+T_{MOD}+ (%)	11	49	36
	A+T_{HIGH}+ (%)	1	9	35

Total n = 475. *CU* Cognitively Unimpaired; *MCI* Mild Cognitive Impairment; *SD* Standard deviation; *IQR* interquartile range; *MMSE* mini-mental state examination; *CDR-SoB* clinical dementia rating sum of boxes; *MK6240* $SUVR_{MetaT}$ Tau ^{18}F-MK6240 SUVR estimated in a standard meta-temporal composite region, comprising entorhinal cortex, parahippocampus, amygdala, inferior temporal, fusiform, and middle temporal cortex ROI; *Aβ PET positive* based on a Centiloid threshold of 25 CL; *A-T-* amyloid negative and tau negative; *A+T-* amyloid positive and tau negative; *A+*T_{MTL}*+* amyloid positive with tau uptake limited to medial temporal region; *A+*T_{MOD}*+* amyloid positive with moderate tau uptake in temporo-parietal region; *A+*T_{HIGH}*+* amyloid positive with high tau uptake in temporo-parietal region.

Supplementary Table 3. The *p*-values and Cohen's *d* effect sizes for differences in plasma p217+tau concentration

Comparisons	Adjusted *p* values	Cohen's *d*	Median fold change
A-T- *vs*. A+T-	<0.001	1.3	1.9
A-T- *vs*. A+T_{MTL}+	<0.001	2.0	2.2
A-T- *vs*. A+T_{MOD}+	<0.001	2.3	3.3
A-T- *vs*. A+T_{HIGH}+	<0.001	3.8	5.3
A+T- *vs*. A+T_{MTL}+	0.181	0.4	1.2
A+T- *vs*. A+T_{MOD}+	<0.001	1.2	1.8
A+T- *vs*. A+T_{HIGH}+	<0.001	2.2	2.8
A+T_{MTL}+ *vs*. A+T_{MOD}+	0.016	0.9	1.5
A+T_{MTL}+ *vs*. A+T_{HIGH}+	<0.001	1.6	2.4
A+T_{MOD}+ *vs*. A+T_{HIGH}+	0.016	1.1	1.6

Multiple comparison of group p217+tau means, using Dunn's test. *A-T-* amyloid negative and tau negative; *A+T-* amyloid positive and tau negative; *A+T_{MTL}+* amyloid positive with tau uptake limited to medial temporal region; *A+T_{MOD}+* amyloid positive with moderate tau uptake in temporo-parietal region; *A+T_{HIGH}+* amyloid positive with high tau uptake in temporo-parietal region.

Supplementary Table 4. Statistics of the ROC analysis to predict disease stage using p217+tau Youden threshold, *CU and CI participants combined*

	AUC	Youden threshold	Sensitivity	Specificity	PPV	NPV
Model 1	0.92 [0.90 - 0.94]	99.38 [89.75 - 102.45]	0.87 [0.83 - 0.91]	0.84 [0.78 - 0.88]	0.89 [0.85 - 0.92]	0.81 [0.77 - 0.87]
Model 2	0.92 [0.90 - 0.94]	168.04 [131.83 - 177.38]	0.77 [0.73 - 0.90]	0.91 [0.80 - 0.95]	0.84 [0.71 - 0.89]	0.88 [0.85 - 0.93]
Model 3	0.91 [0.88 - 0.95]	205.43 [173.18 - 257.86]	0.89 [0.79 - 0.97]	0.82 [0.75 - 0.9]	0.33 [0.25 - 0.47]	0.99 [0.98 - 1.0]

Values reported with bootstrapped 95% confidence intervals, shown in square brackets. Model 1: A-T- vs. A+T-/A+T_{MTL}+/A+T_{MOD}+/A+T_{HIGH}+. PPV and NPV reported at A+ observed prevalence of 60%. The reported AUC value was slightly higher and Youden's index threshold lower than previously reported for this p-tau assay when discriminating based on Aβ status (which was based on cohort 1 p217+tau data) [4]. This is attributed to the cohort 2 differences due to between-lab variations (for details on cohort 1 and cohort 2, see Methods). It is less likely to be due to exclusion of A-T+ individuals from the current study, as repetition of this analysis, with the 31 A-T+ included, yielded an AUC of 0.91 [0.89 - 0.93] and Youden threshold of 99.38 fg/ml [89.75 - 126.68].

Model 2: A-T-/A+T-/A+T_{MTL}+ vs. A+T_{MOD}+/A+T_{HIGH}+. PPV and NPV reported at A+T_{MOD}+/A+T_{HIGH}+ observed prevalence of 36%.

Model 3: A-T-/A+T-/A+T_{MTL}+/A+T_{MOD}+ vs. A+T_{HIGH}+. PPV and NPV reported at A+T_{HIGH}+ observed prevalence of 9%.

A-T- amyloid negative and tau negative; *A+T-* amyloid positive and tau negative; *A+T_{MTL}+* amyloid positive with tau uptake limited to medial temporal region; *A+T_{MOD}+* amyloid positive with moderate tau uptake in temporo-parietal region; *A+T_{HIGH}+* amyloid positive with high tau uptake in temporo-parietal region; *PPV* positive predictive value; *NPV* negative predictive value.

Supplementary Table 5. Statistics of the ROC analysis to predict disease stage using p217+tau Youden threshold, *in CI participants*

	AUC	Youden threshold	Sensitivity	Specificity	PPV	NPV
Model 1	0.93 [0.89 - 0.96]	126.68 [94.83 - 149.88]	0.83 [0.69 - 0.95]	0.86 [0.79 - 1.0]	0.97 [0.96 - 1.0]	0.48 [0.34 - 0.74]
Model 2	0.89 [0.86 - 0.93]	177.38 [168.04 - 179.39]	0.78 [0.74 - 0.86]	0.92 [0.85 - 0.96]	0.94 [0.89 - 0.97]	0.72 [0.66 - 0.81]
Model 3	0.84 [0.78 - 0.89]	230.29 [205.43 - 299.52]	0.84 [0.69 - 0.95]	0.71 [0.64 - 0.88]	0.39 [0.31 - 0.58]	0.95 [0.92 - 0.98]

Values reported with bootstrapped 95% confidence intervals, shown in square brackets. Model 1: A-T- *vs.* A+T-/A+T_{MTL}+/A+T_{MOD}+/A+T_{HIGH}+. PPV and NPV reported at A+ observed prevalence of 84%.

Model 2: A-T-/A+T-/A+T_{MTL}+ *vs.* A+T_{MOD}+/ A+T_{HIGH}+. PPV and NPV reported at A+T_{MOD}+/A+T_{HIGH}+ observed prevalence of 62%.

Model 3: A-T-/A+T-/A+T_{MTL}+/A+T_{MOD}+ *vs.* A+T_{HIGH}+. PPV and NPV reported at A+T_{HIGH}+ observed prevalence of 18%.

A-T- amyloid negative and tau negative; *A+T-* amyloid positive and tau negative; *A+*T_{MTL}*+* amyloid positive with tau uptake limited to medial temporal region; *A+*T_{MOD}*+* amyloid positive with moderate tau uptake in temporo-parietal region; *A+*T_{HIGH}*+* amyloid positive with high tau uptake in temporo-parietal region; *PPV* positive predictive value; *NPV* negative predictive value.

Supplementary Table 6. Discrimination between only two stages at a time, *CU and CI participants combined*

	AUC	Youden threshold	Sensitivity	Specificity	PPV	NPV
A+T- *vs.* A+T_{MTL}+	0.62 [0.53 - 0.72]	116.07 [88.03 – 160.0]	0.74 [0.50 - 1.00]	0.49 [0.28 - 0.77]	0.35 [0.26 - 0.49]	0.83 [0.78 - 1.0]
A+T_{MTL}+ *vs.* A+T_{MOD}+	0.76 [0.70 - 0.83]	178.91 [168.04 – 200.0]	0.68 [0.58 - 0.77]	0.84 [0.73 - 0.96]	0.95 [0.91 - 0.99]	0.39 [0.30 - 0.51]
A+T_{MOD}+ *vs.* A+T_{HIGH}+	0.77 [0.70 - 0.84]	271.34 [230.29 – 330.0]	0.70 [0.59 - 0.90]	0.73 [0.57 - 0.87]	0.47 [0.35 - 0.63]	0.88 [0.84 - 0.95]

Values reported with bootstrapped 95% confidence intervals, shown in square brackets. To discriminate between only two biological PET stages at a time, the sample sizes were smaller, thus we performed this analysis only on the combined CU and CI participants. The AUC values were expectedly lower when using plasma p217+tau to discriminate between only two stages at a time. Values reported with bootstrapped 95% confidence intervals, shown in square brackets. *A+T-* amyloid positive and tau negative; $A+T_{MTL}+$ amyloid positive with tau uptake limited to medial temporal region; $A+T_{MOD}+$ amyloid positive with moderate tau uptake in temporo-parietal region; $A+T_{HIGH}+$ amyloid positive with high tau uptake in temporo-parietal region.

Supplementary Figure 1. ROC analysis for group-level PET staging (including MMSE)

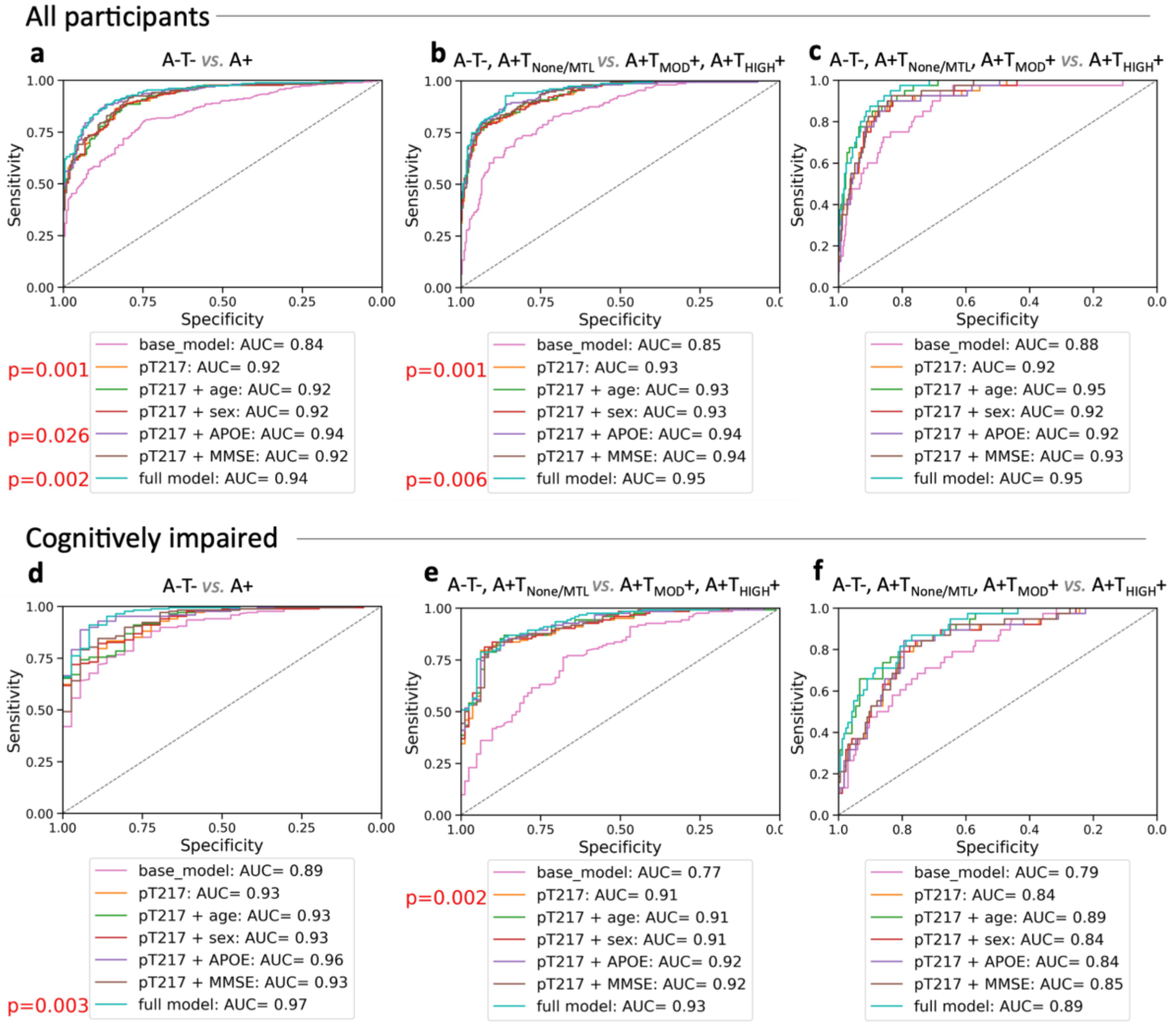

ROC analysis for group-level PET staging, for **a** all participants (n = 443, smaller sample size than Figure 2a due to missing MMSE data), and **b** the cognitively impaired group (n = 203, smaller sample size than Figure 2b due to missing MMSE data). Base model includes age, sex, Apolipoprotein E (*APOE*) ε4 status and MMSE. Full model includes p217+tau, age, sex, *APOE* ε4 status and MMSE. *pT217* plasma p217+tau; *MMSE* Mini-Mental State Examination; *AUC* area under the receiver operating characteristic curve. *A-T-* amyloid negative and tau negative; *A+T*$_{None/MTL}$ amyloid positive and tau negative or amyloid positive with tau uptake limited to medial temporal region; *A+T*$_{MOD}$*+* amyloid positive with moderate tau uptake in temporo-parietal region; *A+T*$_{HIGH}$*+* amyloid positive with high tau uptake in temporo-parietal region. Significant p values from DeLong test are presented for comparison of the model with p217+tau only, to the base model, along with for comparison of all models (except the base model) to the model with p217+tau only (corrected for multiple comparisons).

Supplementary Figure 2. Three different thresholds for discriminating the A+T

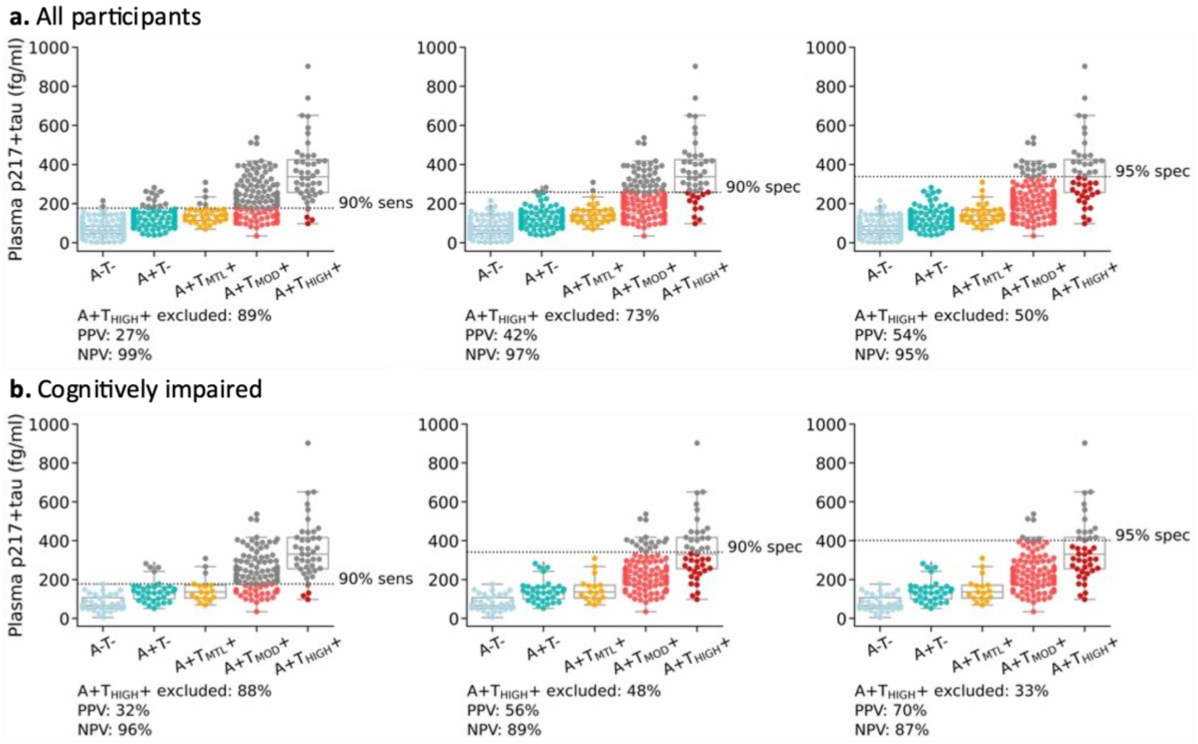

Discrimination of A+T_{HIGH}+ biological PET stage using thresholds set at 90% sensitivity, 90% specificity or 95% specificity, for **a** all participants (n = 475), and **b** the cognitively impaired group (n = 227). The error bars in the boxplots represent 1.5 times the interquartile range (IQR) above and below the upper (75th percentile) and lower (25th percentile) quartiles. If the purpose of screening was to exclude the A+T_{HIGH}+ stage, 90% sensitivity threshold would provide a better cut-off point, however more than half of the A+T_{MOD}+ group would also be screened out. If the purpose of screening was to exclude as many A+T_{HIGH}+ as possible while retaining the majority of A+T_{MOD}+, a threshold set at 95% specificity may be more suitable. *A-T-* amyloid negative and tau negative; *A+T-* amyloid positive and tau negative; *A+T_{MTL}+* amyloid positive with tau uptake limited to medial temporal region; *A+T_{MOD}+* amyloid positive with moderate tau uptake in temporo-parietal region; *A+T_{HIGH}+* amyloid positive with high tau uptake in temporo-parietal region; *90% sens* threshold set at 90% sensitivity for discriminating A+T_{HIGH}+; *90% spec* threshold set at 90% specificity for discriminating A+T_{HIGH}+; *95% spec* threshold set at 95% specificity for discriminating A+T_{HIGH}+; *PPV* positive predictive value; *NPV* negative predictive value.
