# Supplementary material for: Alzheimer’s disease biological PET staging using plasma p217+tau
Source: Commun Med (Lond). 2025 Feb 27;5:53. doi: 10.1038/s43856-025-00768-z (PMC11868538; doi:10.1038/s43856-025-00768-z)
Supplement: Supplementary file 3 — Description of Additional Supplementary Files [file 43856_2025_768_MOESM3_ESM.pdf]

Description of Additional Supplementary Files

File name: Supplementary Data 1-3

File description: The source data for Figures 1-3
