# Supplementary material for: Alzheimer’s disease biological PET staging using plasma p217+tau
Source: Commun Med (Lond). 2025 Feb 27;5:53. doi: 10.1038/s43856-025-00768-z (PMC11868538; doi:10.1038/s43856-025-00768-z)
Supplement: Supplementary file 5 — Reporting Summary [file 43856_2025_768_MOESM5_ESM.pdf]

nature portfolio

Corresponding author(s): Prof. Christopher C. Rowe

Last updated by author(s): Dec 9, 2024

Reporting Summary

Nature Portfolio wishes to improve the reproducibility of the work that we publish. This form provides structure for consistency and transparency in reporting. For further information on Nature Portfolio policies, see our Editorial Policies and the Editorial Policy Checklist.

Statistics

For all statistical analyses, confirm that the following items are present in the figure legend, table legend, main text, or Methods section.

n/a	Confirmed	
☐	☒	The exact sample size (*n*) for each experimental group/condition, given as a discrete number and unit of measurement
☐	☒	A statement on whether measurements were taken from distinct samples or whether the same sample was measured repeatedly
☐	☒	The statistical test(s) used AND whether they are one- or two-sided<br>*Only common tests should be described solely by name; describe more complex techniques in the Methods section.*
☐	☒	A description of all covariates tested
☐	☒	A description of any assumptions or corrections, such as tests of normality and adjustment for multiple comparisons
☐	☒	A full description of the statistical parameters including central tendency (e.g. means) or other basic estimates (e.g. regression coefficient) AND variation (e.g. standard deviation) or associated estimates of uncertainty (e.g. confidence intervals)
☐	☒	For null hypothesis testing, the test statistic (e.g. *F*, *t*, *r*) with confidence intervals, effect sizes, degrees of freedom and *P* value noted<br>*Give P values as exact values whenever suitable.*
☒	☐	For Bayesian analysis, information on the choice of priors and Markov chain Monte Carlo settings
☒	☐	For hierarchical and complex designs, identification of the appropriate level for tests and full reporting of outcomes
☐	☒	Estimates of effect sizes (e.g. Cohen's *d*, Pearson's *r*), indicating how they were calculated

Our web collection on statistics for biologists contains articles on many of the points above.

Software and code

Policy information about availability of computer code

Data collection	No software was used.
Data analysis	All analyses were completed using Python 3.9.13 (main, Aug 25 2022). Libraries include:<br>numpy<br>pandas<br>seaborn<br>matplotlib<br>sklearn<br>scipy<br>statsmodels<br>scikit

For manuscripts utilizing custom algorithms or software that are central to the research but not yet described in published literature, software must be made available to editors and reviewers. We strongly encourage code deposition in a community repository (e.g. GitHub). See the Nature Portfolio guidelines for submitting code & software for further information.

Data

Policy information about availability of data

All manuscripts must include a data availability statement. This statement should provide the following information, where applicable:

- Accession codes, unique identifiers, or web links for publicly available datasets
- A description of any restrictions on data availability
- For clinical datasets or third party data, please ensure that the statement adheres to our policy

The anonymized ADNeT data reported in the manuscript are available from the corresponding author upon reasonable request, from a qualified academic investigator for the purpose of replicating the results presented in the article. Access to the deidentified AIBL data can be achieved by submitting an expression of interest (EOI) to AIBL via www.aibl.csiro.au.

Human research participants

Policy information about studies involving human research participants and Sex and Gender in Research.

Reporting on sex and gender	In our manuscript, we have reported percentage of participants that were female vs male in each studied group. This refers to their self-reported sex. We have included this variable in our analysis as covariates or additional predictors in our ROC models. The primary purpose of our analysis has been to discriminate between the biological PET stages of AD, no matter what clinical diagnosis or gender/sex. In our ROC models, additional information about being Female vs Male did not improve the accuracy of the models and therefore did not warrant more detailed investigation of the effect of sex on the studied construct.
Population characteristics	Demographic characteristics of the participants are presented in Table 2 and Supplementary Table 2. Participants included 248 Cognitively Unimpaired, 144 with Mild Cognitive Impairment and 83 with dementia (total n = 475). No significant sex differences were observed among the groups.
Recruitment	Participants were recruited as a part of the Australian Imaging Biomarkers and Lifestyle flagship study of aging (AIBL) and Australian Dementia Network (ADNeT) studies based on established protocols by these two studies.
Ethics oversight	Approval was obtained from institutional ethics review committees for the AIBL and ADNeT studies and written informed consent was obtained from all participants.

Note that full information on the approval of the study protocol must also be provided in the manuscript.

Field-specific reporting

Please select the one below that is the best fit for your research. If you are not sure, read the appropriate sections before making your selection.

☒ Life sciences ☐ Behavioural & social sciences ☐ Ecological, evolutionary & environmental sciences

For a reference copy of the document with all sections, see nature.com/documents/nr-reporting-summary-flat.pdf

Life sciences study design

All studies must disclose on these points even when the disclosure is negative.

Sample size	Our study included a large sample of 475 participants. This was the sample size available from two large-scale studies of AIBL and ADNeT with data from participants with matched 18F-MK6240 tau PET, 18F-NAV4694 93 Aβ PET and plasma p217+tau. We did not perform a sample size calculation for this study. We aimed to maximize the sample size by pooling data across AIBL and ADNeT cohorts. We used the following inclusion criteria: All participants were i) either cognitively unimpaired, defined by neuropsychological test scores within the normative range for their age, or had mild cognitive impairment or dementia, ii) had amyloid PET available to determine amyloid status, iii) had tau PET scan to determine the biological PET stages and iv) had their plasma samples assayed for p217+tau.
Data exclusions	The total dataset included 506 participants with blood results as well as PET data. Of the 506 participants, 31 who were Amyloid negative (A-) but tau positive (T+) in one or all three brain Regions Of Interest did not fit within any of the above NIA-AA defined categories and were excluded from subsequent analyses. Among these, 11 had mildly elevated MTL binding and were postulated to have primary age related tauopathy (PART) but 9 were positive on quantification in MTL and temporo-parietal. This proportion of the total study sample of ~2% as having clearly positive neocortical tau PET but negative amyloid PET is consistent with our previous report (see Supplementary Table 1 for demographic characteristics of the excluded participants).<br><br>For the analysis pertaining regression curves, given that there were only nine participants with p217+tau values above 500 fg/ml, with a range from 507 to 902 fg/ml (6 of whom were A+THIGH+ and 3 were A+TMOD+) and considering the limited statistical power to estimate the line of best fit, we restricted our regression analyses to plasma p217+tau concentrations between 0 and 500 fg/ml, just for this specific analysis.

Replication	We pooled the AIBL and ADNeT data to maximize the sample size per clinical group. Therefore, we did not replicate the findings in separate cohorts.
Randomization	Biological PET stages were defined based on the Revised Criteria for Diagnosis and Staging of Alzheimer's Disease (2024): Initial (A+T-), Early (A+TMTL+), Intermediate (A+TMOD+), and Advanced (A+THIGH+).
Blinding	Amyloid and tau PET analyses were performed by individuals who were blinded to the clinical data or aims of the study.

Reporting for specific materials, systems and methods

We require information from authors about some types of materials, experimental systems and methods used in many studies. Here, indicate whether each material, system or method listed is relevant to your study. If you are not sure if a list item applies to your research, read the appropriate section before selecting a response.

Materials & experimental systems

n/a	Involved in the study
☒	☐ Antibodies
☒	☐ Eukaryotic cell lines
☒	☐ Palaeontology and archaeology
☒	☐ Animals and other organisms
☒	☐ Clinical data
☒	☐ Dual use research of concern

Methods

n/a	Involved in the study
☒	☐ ChIP-seq
☒	☐ Flow cytometry
☒	☐ MRI-based neuroimaging
