# Supplementary material for: Alzheimer’s disease biological PET staging using plasma p217+tau
Source: Commun Med (Lond). 2025 Feb 27;5:53. doi: 10.1038/s43856-025-00768-z (PMC11868538; doi:10.1038/s43856-025-00768-z)
Supplement: Supplementary file 1 — Transparent Peer Review file [file 43856_2025_768_MOESM1_ESM.pdf]

nature portfolio

Peer Review File

Alzheimer’s Disease biological PET staging using plasma p217+tau

Corresponding Author: Professor Christopher Rowe

This file contains all reviewer reports in order by version, followed by all author rebuttals in order by version.

Version 0:

Reviewer comments:

Reviewer #1

(Remarks to the Author)
COMMSMED-23-0953-T.
Alzheimer’s Disease biological PET staging using plasma pTau217+tau
Feizpour et al.

In this paper, the authors aim to determine if plasma pTau217 can replace Ab and Tau PET imaging in the staging of individuals with Alzheimer’s disease (AD) and different levels of tau pathology for clinical trials and disease modifying therapies. While several prior studies have demonstrated that pTau217 levels increase stepwise when stratified by amyloid and tau PET status and/or with increasing Braak stages, less information is available about the suitability of pTau217 to differentiate between different stages and if cutpoints with adequate accuracy can be established. The results show that pTau217 perform well in separating high contrast groups (A-T-/Initial/Early vs Intermediate/High) while the discriminatory ability to separate adjacent groups (A+T- vs Initial or Intermediate vs High) is less convincing. The logistic regression probability scores presented in Figure 3 clearly show the overlap in pTau217 levels between the different PET stages and the challenge in using pTau217 to stage on an individual level.

A main question is why the authors choose to focus on the Youden index when establishing cutpoints. While identifying an optimal cutpoint based on the combination of sensitivity and specificity, the Youden index is not adapted to the intended use of the cutpoint and it may be better to define different level probability cutpoints based on the desired sensitivity and specificity of the intended use in line with Brum et al. (Nat Aging 2023). That is, if the cutpoint will be used to ascertain that subjects with high tau burden are excluded from a potential clinical trial as suggested in the manuscript, it may be better to identify cutpoints with high sensitivity and a high PPV to detect high tau burden, etc.

While Figure 3 clearly illustrates the overlap in pTau217 between the different PET stages as mentioned above, it is difficult to understand what the probability is for a particular outcome at the different pTau217 levels, and it may have been beneficial with a table outlining the probability levels for the different sequential pTau217 bands.

The different models used for the ROC analysis included age, gender, and APOE status, but did not consider cognitive status. With a goal to use pTau217 to stage individuals for inclusion in clinical trials, it would have been relevant to determine if pTau217 levels add to a base model including cognitive status.

Minor questions:
1. What were the diagnoses of the non-AD participants?
2. What was the precision of the pTau217 assay?

Taken together, while using appropriate methods and an adequate sample size were used, the novel findings in the manuscript are relatively limited and the applicability of the cutpoints identified as described are uncertain for the intended use. The manuscript would furthermore benefit from including more of the existing literature on pTau217 in relation to Ab and Tau burden on PET besides the few papers currently discussed to better reflect the current thinking around these questions.

Reviewer #2

(Remarks to the Author)
The authors aimed at investigating the value of Janssen plasma p217+tau Simoa assay for predicting amyloid PET positivity (A+) using 18F-NAV4694 Aβ PET and for separating between different Tau PET stages using 18F-MK6240 tau PET in 248 cognitively unimpaired and 227 cognitively impaired individuals from the AIBL and ADNeT cohorts.
AUCs, sensitivity and specificities were calculated between different groups based on amyloid PET positivity and Tau PET staging as proposed by NIA-AA. In addition, individual prediction of a specific tau stage has been calculated.

Strengths:
Very large sample size;
Highly characterized subjects
Clinically important research question
State-of-the-art methodology.

Major concerns:
1. While the conclusions of group level analyses are supported by results, assessment of individual prediction in the abstract is too optimistic (line 49, "good discrimination"), assessment in the discussion section is much more appropriate (l 406 confident staging ... is limited). Hence, the authors are requested to soften their assessment in the abstract.

2. The authors have decided to exclude certain patients from analyses who did not fit into their model (>5%, line 158), or had high plasma values (>1% l 183). While understandable from a methodological point of view this approach is not compatible with their aim to recruit for a clinical trial (l 353). The authors are requested to explicitly discuss these post-hoc exclusions and that these procedures have probably lead to an overestimation of accuracy measures.

3. While being very precise on PET technology, information on the plasma pTau217 assay which is not approved for diagnostic purposes is sparse. The authors quoted one previous study in which substantial difficulties in accurately measuring pTau217 were reported. Therefore, the authors are requested to provide the main procedures of the method sheet of the assay as a supplement and to report basic characteristics of the assay: measured in quadruplicate? maximum and mean CVs and their impact on using the assay for individual testing?; LLOQ?. Interestingly, the previous study reported a LLOQ a cutoff of 37 fg/mL, Figure 1 suggests lower values, please also comment.

4. Limitations: The authors are requested to discuss that optimized cutoffs have been calculated within the sample and no independent assessment of the cutoffs have been performed to prospectively assess accuracy measures.

Reviewer #3

(Remarks to the Author)
Brief summary of the manuscript
The authors describe the use of plasma p217+tau (an immunoassay on the Simoa platform, targeting p-tau217 with some cross-reactivity to p-tau212) in discriminating AT stages defined by amyloid and tau PET, in a total of 475 participants in the AIBL and ADNeT studies (comprising 248 cognitively unimpaired and 227 cognitively impaired individuals). The overall prevalence of amyloid PET positivity (defined as >25 Centiloid) was about 60% and the prevalence of TMOD/HIGH+ was 36%. The main contribution of this work that advances the field is to examine the utility of various cut-points for discriminating different AT contrasts as defined by amyloid PET, and to attempt individual level prediction (though as demonstrated in this work the latter is limited when using this biomarker on its own).

Overall impression of the work
The paper is very well written; the research questions and most of the methodology used to answer them are clearly detailed. However the participant selection from the main cohorts is not described at all, and the only reference given regarding the recruitment to the original cohorts refers to AIBL and not ADNeT. As the utility of plasma p-tau217 testing is highly contingent upon the prior likelihood of AD in the population tested (which can be indexed as the prevalence of A+ and Tmod/high in that population), this is a fundamental point that needs to be detailed in both the abstract and the results, and will allow the reader to ascertain how representative the study sample is of the cohorts from which it was drawn, and any limitations in generalising to other settings where different prevalence may be seen. See specific recommendations below.

Specific comments, with recommendations for addressing each comment
The abstract would benefit from clearly stating the prevalence of A+ and of THIGH+ at least, and qualifying “good discrimination” at the individual level (line 50)

Introduction
Line 67 – suggest clarifying “similar performance to p-tau217” to “similar performance to plasma p-tau217” as currently this line could be interpreted as p-tau217 measured in CSF.

Methods
This paper would benefit from a flow chart showing how the final number of participants/samples was derived from the parent cohorts. Please provide the inclusion and exclusion criteria applied to arrive at the final sample number, as well as a

reference, or further detail in the supplementary information, regarding the ADNeT cohort in particular. Overall from looking at the reference provided for AIBL it is apparent that individuals with MCI and AD dementia were recruited from memory clinics but there was a mix of memory non-complainers and memory complainers among those classified as cognitively normal, and it would be helpful to know how they were recruited.
It is interesting to note (from Results table 2) that those with A+THIGH+ were significantly younger than those in other groups and it would be helpful to know why that may have occurred at recruitment level (e.g. is there a weighting toward younger individuals with dementia being recruited rather than older individuals, and how that compares across the cohorts)?

It would be beneficial to include some detail on the actual testing of the samples eg singlicate vs duplicate testing, and how harmonization across testing carried out in 2 laboratories was ensured beyond stating that “assay scales measured from both laboratories were comparable” (line 141-142). Was a bridging study or at least sharing of common control samples employed across the two laboratories/two sets of quantifications? Were there different reagent batches used even within each laboratory’s quantifications? These details are interesting to those in the field as we move toward testing the performance of these types of assay in the real world across laboratories and across reagent batches.

Statistical methods
Looking at the dot plot overlaying the box plot on Figure 1, it is unlikely that the distributions of plasma p217+tau within each AT group met the criteria for parametric testing with ANOVA and Tukey HSD (namely there is a skew more evident in some groups, and clear inhomogeneity of variances across the groups) so it is likely that a non-parametric method of comparison would be more appropriate (eg Kruskal-Wallis ANOVA followed by post hoc Dunn’s test) and the more appropriate summary effect size would be median fold change rather than Cohen’s D. I would suggest that these tests and values at least be included in supplementary information as an alternative.

Results
The results at group level are framed in terms of AUC and the sensitivity, specificity, PPV and NPV obtained using single cut-points at Youden’s index. However an increasingly favoured approach for p-tau217 is to use two cutpoints that fix sensitivity and specificity (eg at 95% for both), and employ a grey zone – see Brum et al. 2023 (DOI: 10.1038/s43587-023-00471-5). It would be beneficial to include a similar exploration of the PPV and NPV obtained using this approach.

Lines 305-317 detail a sub-analysis within the group of individuals who have p217+tau >177.4 fg/ml; to determine the utility of a further cut-point based on ROC analysis within this sub-group, in discriminating those with THiGH+ from those in other disease stages. It would be helpful to know the size of this analysis group and the split of THIGH+ vs Tother within it, as if the T groups are significantly imbalanced in terms of numbers, AUC may overstate the discriminant value of this biomarker at this end of the spectrum of values.

While the individual level predictions of disease stage based on the logistic regressions (p217+tau as either continuous predictor or in 80 fg/ml bands) are interesting, they demonstrate the difficulty in predicting disease stage beyond the extremes of A-T- and A+THIGH+ based on this biomarker alone. While the authors state that this means that “One instead should consider an individual participant’s
likelihood for belonging to each of the stages based on their p217+tau level as illustrated by Figure 3.”, it is difficult to see how a clinician would interpret for example a p217+tau value of 160 pg/ml in a cognitively impaired individual which according to figure 3B would confer equal probability of A+/Tnon/MTL+ and A+TMod+ (each around 40%) and equal lower probability of A-T- and A+THIGH+ (each around 5-10%). This should be recognized as a limitation in the use of individual level prediction that may or may not be improved by incorporation of other individual level factors (as the authors recommend) into risk prediction models.

Version 1:

Reviewer comments:

Reviewer #2

(Remarks to the Author)
The authors have addressed all concerns. Thanks a lot.
I recommend to publish the mmanuscript in its current form incoorperating the following minor concern.

Minor Concern:
Please state in the limitation section: CVs were up to 20%. This uncertainty would need to be considered when using pTau217 as a diagnostic test.

Reviewer #3

(Remarks to the Author)
I am satisfied that the authors have adequately addressed the concerns of both myself and the other reviewers in the revised manuscript.

Open Access This Peer Review File is licensed under a Creative Commons Attribution 4.0 International License, which permits use, sharing, adaptation, distribution and reproduction in any medium or format, as long as you give appropriate credit to the original author(s) and the source, provide a link to the Creative Commons license, and indicate if changes were made.
In cases where reviewers are anonymous, credit should be given to 'Anonymous Referee' and the source.
The images or other third party material in this Peer Review File are included in the article's Creative Commons license, unless indicated otherwise in a credit line to the material. If material is not included in the article's Creative Commons license and your intended use is not permitted by statutory regulation or exceeds the permitted use, you will need to obtain permission directly from the copyright holder.
To view a copy of this license, visit https://creativecommons.org/licenses/by/4.0/

Reviewers' comments:

Reviewer #1 (Remarks to the Author):

1. A main question is why the authors choose to focus on the Youden index when establishing cutpoints. While identifying an optimal cutpoint based on the combination of sensitivity and specificity, the Youden index is not adapted to the intended use of the cutpoint and it may be better to define different level probability cutpoints based on the desired sensitivity and specificity of the intended use in line with Brum et al. (Nat Aging 2023). That is, if the cutpoint will be used to ascertain that subjects with high tau burden are excluded from a potential clinical trial as suggested in the manuscript, it may be better to identify cutpoints with high sensitivity and a high PPV to detect high tau burden, etc.
We have revised the Results section to reflect this addition :
"We also evaluated thresholds chosen for 90% sensitivity, 90% specificity and 95% specificity for A+T$_{HIGH}$+ (see Supplementary Figure 1) but no threshold was able to provide both satisfactory sensitivity and PPV (e.g. in CI a 90% sensitivity threshold gave PPV of only 32%; a 95% specificity threshold gave PPV of 70% with only 33% sensitivity). Thus adjusting the threshold to improve the PPV resulted in substantially reduced sensitivity due to the high degree of overlap with Intermediate stage AD, as can be seen in Figure 1."

Added supplementary Figure 1:

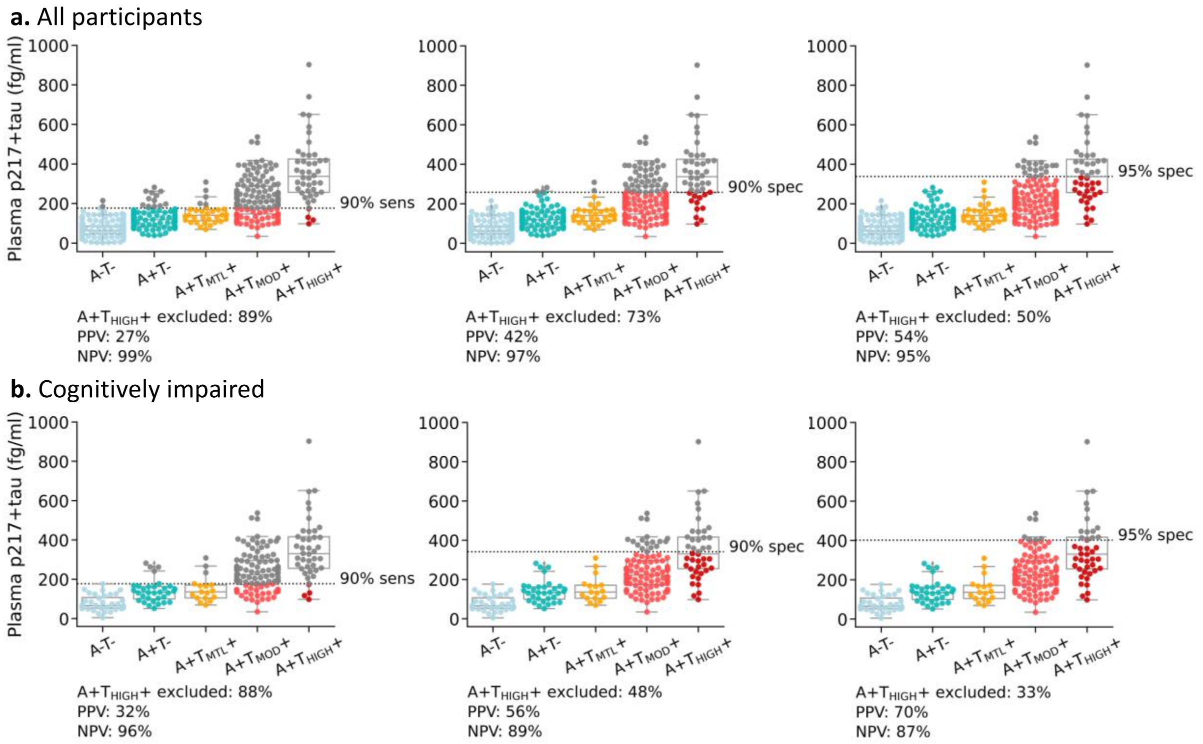

2. While Figure 3 clearly illustrates the overlap in pTau217 between the different PET stages as mentioned above, it is difficult to understand what the probability is for a particular outcome at the different pTau217 levels, and it may have been beneficial with a table outlining the probability levels for the different sequential pTau217 bands.
Thanks for this suggestion. We have added two tables to Figure 3 addressing this

comment. Figure 3 legend is revised to reflect this revision: “The tables below the figures outline the probability scores for the different sequential p217+tau levels”.

Revised Figure 3:

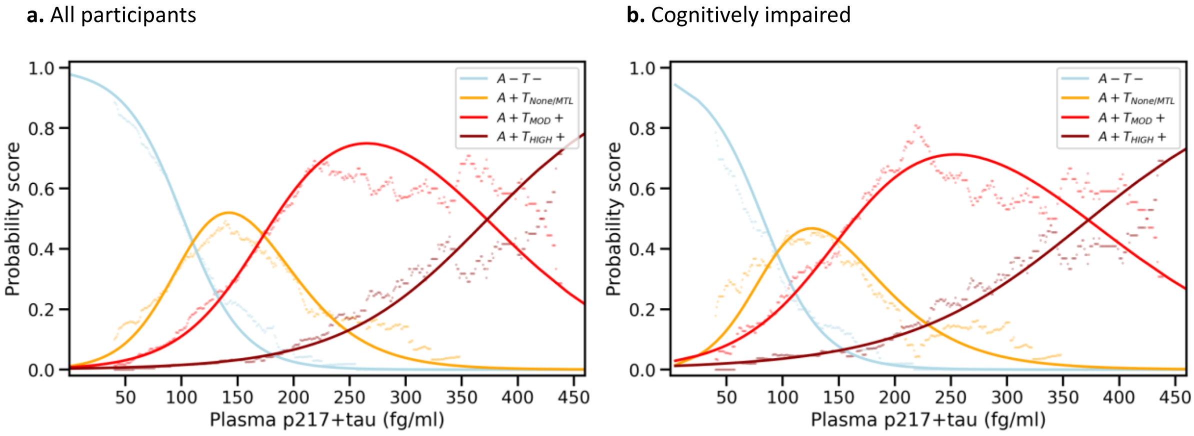

	0.88	0.52	0.14	0.03	0	0	0	0
	0.09	0.36	0.51	0.32	0.12	0.04	0.01	0
	0.03	0.11	0.31	0.59	0.74	0.72	0.57	0.4
	0.01	0.02	0.03	0.07	0.13	0.24	0.42	0.6

	0.77	0.34	0.08	0.01	0	0	0	0
	0.13	0.41	0.43	0.24	0.1	0.04	0.01	0.01
	0.08	0.21	0.43	0.63	0.71	0.67	0.55	0.42
	0.02	0.04	0.06	0.11	0.18	0.29	0.43	0.57

3. The different models used for the ROC analysis included age, gender, and APOE status, but did not consider cognitive status. With a goal to use pTau217 to stage individuals for inclusion in clinical trials, it would have been relevant to determine if pTau217 levels add to a base model including cognitive status.

   Thanks for this suggestion. Our base model consisted of established risk factors of age, sex and APOE e4 carriership, similar to previous studies such as:
   Jack, C. R. *et al.* Predicting amyloid PET and tau PET stages with plasma biomarkers. *Brain* **146**, 2029-2044 (2023). https://doi.org/10.1093/brain/awad042

   Figure 2 consists of a top panel (Figure 2a. in the revised manuscript) which includes all participants (CU, MCI and dementia), while the bottom panel (Figure 2b. in the revised manuscript) only includes participants with MCI or dementia, thus cognitive stage is taken into consideration to some degree.

   However, to further assess your suggestion, we added a supplementary Figure 2 where MMSE is added to the base model, p217+tau and the full model. Adding MMSE had no effect on the full model AUC though did substantially improve the base model. Consequently the addition of MMSE is only briefly mentioned in the Results section “Adding MMSE score to the full model did not assist AD staging (see Supplementary Figure 2).”

4. What were the diagnoses of the non-AD participants?
   Inclusion/exclusion criteria for AIBL/ADNeT were weighted towards recruitment of possible AD. Therefore, all participants in AIBL/ADNeT AD dementia subcohorts met “clinical” criteria for either possible or probable AD at enrolment, and the diagnosis was not reviewed after PET results. Participants with definite features of other neurodegenerative diseases were excluded.

5. What was the precision of the pTau217 assay?
Triana-Baltzer et al. (2021) state:
"Here we describe a highly sensitive, quantitative, robust, and scalable method for measuring p217+tau in human plasma. The method has a dynamic range of 2 to 10,000 fg/mL and reports all plasma samples (including healthy controls) in this linear range. The method has good inter- and intra-test precision (< 20%) and dilution linearity in the range of quantifiable signal (2–6x)". Page 11 of Triana-Baltzer et al., reference 22 in the manuscript.

6. Taken together, while using appropriate methods and an adequate sample size were used, the novel findings in the manuscript are relatively limited and the applicability of the cutpoints identified as described are uncertain for the intended use. The manuscript would furthermore benefit from including more of the existing literature on pTau217 in relation to Ab and Tau burden on PET besides the few papers currently discussed to better reflect the current thinking around these questions.
We have revised the manuscript to address the points raised by the reviewer.

At AAIC 2023, the Alzheimer's Association Workgroup introduced a revised diagnostic and staging criteria for Alzheimer's disease. This scheme was officially published in July 2024 (DOI: https://doi.org/10.1002/alz.13859) and is now reference 10 in the manuscript. In our manuscript, originally submitted in December 2023, we applied this novel staging scheme and demonstrated how the plasma biomarker p217+tau discriminates between these stages. The new criteria paper does not define "High tau" so we clearly defined High tau as the top 25% of neocortical tau in amyloid positive CI persons in the AIBL study and reported that this corresponds to 80 on the new Centaur scale for quantification of tau PET. A top 25% threshold was used in the Lilly Phase III donanemab study but we are the first to also adopt this definition and the first to define it for MK6240 tau PET. This underscores the novelty and timeliness of our findings.

We have highlighted this important information by expanding on this point in the methods section: "The Te upper threshold was derived using the method from the Trailblazer-Alz 2 clinical trial[1] by defining High tau (A+T_{HIGH}+) as the top quartile of tau PET results in the Aβ+ MCI/AD participants within the entire AIBL/ADNeT studies who have undergone ^{18}F-MK6240 tau PET imaging (n= 348)".

Additionally, while most existing research focuses on ROC-based analyses to assess and compare the performance of plasma biomarkers at the group level, such as for clinical trial screening, our study recognizes the limitations of this approach in addressing individual-level utility. Therefore, we included a novel strategy (figure 3) to better evaluate the utility of plasma biomarkers for individual patients.

Reviewer #2 (Remarks to the Author):

The authors aimed at investigating the value of Janssen plasma p217+tau Simoa assay for predicting amyloid PET positivity (A+) using 18F-NAV4694 Aβ PET and for separating between different Tau PET stages using 18F-MK6240 tau PET in 248 cognitively unimpaired and 227 cognitively impaired individuals from the AIBL and ADNeT cohorts.
AUCs, sensitivity and specificities were calculated between different groups based on amyloid PET positivity and Tau PET staging as proposed by NIA-AA. In addition, individual prediction of a specific tau stage has been calculated.

Strengths:
Very large sample size;
Highly characterized subjects
Clinically important research question
State-of-the-art methodology.

1. While the conclusions of group level analyses are supported by results, assessment of individual prediction in the abstract is too optimistic (line 49, "good discrimination"), assessment in the discussion section is much more appropriate (l 406 confident staging ... is limited). Hence, the authors are requested to soften their assessment in the abstract.
   We revisited the data and agree that the AUC values mislead the reader on the ability to discriminate individual stages and have now revised the manuscript accordingly.

   Amongst the many changes we have made to clarify this issue are:

   Abstract now includes:
   "Youden p217+tau thresholds gave sensitivity 0.77 [0.73 - 0.90], specificity 0.91 [0.80 - 0.95], PPV 0.84 [0.71 - 0.89] and NPV 0.88 [0.85 - 0.93] for combined Intermediate/Advanced stages. For Advanced stage alone sensitivity was 0.89 [0.79 - 0.97], specificity 0.82 [0.75 - 0.9], NPV 0.99 [0.98 - 1.0] but PPV only 0.33 [0.25 - 0.47].
   Conclusion: In addition to accurately screening for A+ individuals, plasma p217+tau may be useful to identify a combined Intermediate/Advanced stage AD cohort or pre-screen to reduce the tau PET required to identify Advanced stage AD individuals"

   Results include the following under the "Discrimination between A+T_{HIGH}+ and combined A-T-/A+$T_{None/MTL}$/A+T_{MOD}+" sub-heading:
   "In CI the Youden index provided a threshold of 230.3 fg/ml [205.4 - 299.5 fg/ml] giving sensitivity of 0.84 [0.69 - 0.95], and specificity 0.71 [0.64 - 0.88] (Supplementary Table 5). With PPV of 0.39 [0.31 - 0.58] and NPV of 0.95 [0.92 - 0.98], p217+tau effectively excluded Advanced stage AD when below the Youden threshold, but did not accurately predict Advanced stage when above, due to the inclusion of many Intermediate stage AD participants"

   Also added to the results section:
   "We also evaluated thresholds chosen for 90% sensitivity, 90% specificity and 95% specificity for A+T_{HIGH}+ (see Supplementary Figure 1) but no threshold was able to provide both satisfactory sensitivity and PPV (e.g. in CI a 90% sensitivity threshold gave PPV of only 32%; a 95% specificity threshold gave PPV of 70% with only 33%

sensitivity). Thus adjusting the threshold to improve the PPV resulted in substantially reduced sensitivity due to the high degree of overlap with Intermediate stage AD, as can be seen in Figure 1"

In Discussion:
"…, the value of plasma p217+tau appears to lie in the detection of A+ individuals and to reduce the required number of tau PET for trial recruitment by defining a combined Intermediate/Advanced AD stage cohort where tau PET can then be used to separate Advanced from Intermediate stage. Similarly with a 95% NPV for Advanced stage AD when applied across all CI participants, plasma p217+tau may be used to screen out persons unlikely to have Advanced AD, with tau PET then required to separate Advanced AD from lower stages in those above the plasma p217+tau threshold"

"The overlap between the range of plasma p217+tau levels was again apparent in a risk prediction model using p217+tau as the predictor and estimated probability scores for an individuals' absolute risk of falling within a disease stage. With the overlap in likelihood functions for most PET stages, confident staging of an individual with plasma p217+tau alone is limited to very low or very high levels (Figure 3)."

"To conclude, plasma p217+tau performs well for detecting persons with brain amyloid and discriminating those with Intermediate or Advanced stages from those at a lower stage, a useful property to reduce the number of tau PET required when screening for individuals at a particular stage of AD"

2. The authors have decided to exclude certain patients from analyses who did not fit into their model (>5%, line 158), or had high plasma values (>1% l 183). While understandable from a methodological point of view this approach is not compatible with their aim to recruit for a clinical trial (l 353). The authors are requested to explicitly discuss these post-hoc exclusions and that these procedures have probably lead to an overestimation of accuracy measures.
Given that exclusion of the A-T+ group would have the highest impact on Model 1 (A-T- vs A+), we did repeat this analysis including the A-T+ group (A- vs A+), for Model 1, with the results reported in the supplementary material: "Model 1: A-T- vs. A+T-/A+T$_{MTL}$+/A+T$_{MOD}$+/A+T$_{HIGH}$+ … repetition of this analysis, with the 31 A-T+ included, yielded an AUC of 0.91 [0.89 - 0.93] and Youden threshold of 99.38 fg/ml [89.75 - 126.68]". The AUC, excluding A-T+ was only slightly increased from 0.91 to 0.92 [0.90 - 0.94] and the Youden threshold was the same, at 99.38 fg/ml.

We have added the following statement to the Results section: "Including the excluded 31 A-T+ individuals in the A- group (n=506, 56% A+), minimally affected the AUC 0.91 [0.89 - 0.93] vs 0.92 [0.90 - 0.94] and the Youden derived p217+tau threshold was unchanged producing an accuracy of 84%"

Exclusion of nine participants with p217+tau values above 500 fg/ml was inevitable as logistic regression curve fitting could be unreliable in presence of outliers. Please note that these 9 values were included in all other analyses throughout the paper and only removed for curve fitting.

3. While being very precise on PET technology, information on the plasma pTau217 assay which is not approved for diagnostic purposes is sparse. The authors quoted one previous study in which substantial difficulties in accurately measuring pTau217 were reported.
   The cited paper by Triana-Baltzer et al. (2021) reports the basic performance of the plasma p217+tau assay. The substantial difficulties reported in Triana-Baltzer et al. (2021) pertain use of CSF p217+tau assay in its original form in plasma which was not fit for purpose. In this paper, the authors report the development of a new assay (p217+tau assay) that overcame previously reported challenges.

4. Therefore, the authors are requested to provide the main procedures of the method sheet of the assay as a supplement and to report basic characteristics of the assay: measured in quadruplicate? maximum and mean CVs and their impact on using the assay for individual testing?; LLOQ?. Interestingly, the previous study reported a LLOQ a cutoff of 37 fg/mL, Figure 1 suggests lower values, please also comment.

   Triana-Baltzer et al. (2021) state:
   "Here we describe a highly sensitive, quantitative, robust, and scalable method for measuring p217+tau in human plasma. The method has a dynamic range of 2 to 10,000 fg/mL and reports all plasma samples (including healthy controls) in this linear range. The method has good inter- and intra-test precision (< 20%) and dilution linearity in the range of quantifiable signal (2–6x)." Page 11 of Triana-Baltzer et al. (2021) PDF.

   "…, in Palmqvist et al, 25% of HC subjects and 7% of AD subjects had MSD p217tau plasma measurements below limit of detection, while here we show that all HC and AD subjects had Simoa p217+tau plasma measurements in linear range." Page 13 of Triana-Baltzer et al. (2021) PDF.

   All samples in our study were measured in duplicate in a given run. This is added to the Methods section: "The samples were measured in duplicate".

   In our study, we had only one individual (1.20 fg/ml) with p217+tau < LLOD (<2 fg/ml). It is recommended to report those values as 50% of LLOD or in our case 1 pg/ml, as is done for ~1/3 of the C2N values in Schinder et al 2024 (see Results section, Sensitivity and variance of assays) in https://www.medrxiv.org/content/10.1101/2024.06.12.24308839v2).

   In our study, we had 30 individuals with p217+tau < LLOQ (<37fg/ml). For those with pTau217< LLOD, it is recommenced to include those values as long as they have good precision. All the 30 samples <LLOQ had <20% CV. Therefore, they were included in our analyses. This additional information is added to the manuscript: "Of the 506 samples included, 30 samples had p217+tau values below the Lower Limit of Quantification (LLOQ: 37 fg/ml). However, all of these samples had a coefficient of variation (CV) of less than 20% and were therefore included in our analyses".

5. Limitations: The authors are requested to discuss that optimized cutoffs have been calculated within the sample and no independent assessment of the cutoffs have been performed to prospectively assess accuracy measures.
   We have now added this to other limitations stated in the Discussion "Replication of

prevalence-dependent findings (i.e. PPV, NPV, estimated probability scores, **cut-off values**) in a “real-world” memory clinic setting is needed before any of the findings could be applied in clinical practice.”

Reviewer #3 (Remarks to the Author):

Brief summary of the manuscript
The authors describe the use of plasma p217+tau (an immunoassay on the Simoa platform, targeting p-tau217 with some cross-reactivity to p-tau212) in discriminating AT stages defined by amyloid and tau PET, in a total of 475 participants in the AIBL and ADNeT studies (comprising 248 cognitively unimpaired and 227 cognitively impaired individuals). The overall prevalence of amyloid PET positivity (defined as >25 Centiloid) was about 60% and the prevalence of TMOD/HIGH+ was 36%. The main contribution of this work that advances the field is to examine the utility of various cut-points for discriminating different AT contrasts as defined by amyloid PET, and to attempt individual level prediction (though as demonstrated in this work the latter is limited when using this biomarker on its own).The paper is very well written; the research questions and most of the methodology used to answer them are clearly detailed.

However the participant selection from the main cohorts is not described at all, and the only reference given regarding the recruitment to the original cohorts refers to AIBL and not ADNeT. As the utility of plasma p-tau217 testing is highly contingent upon the prior likelihood of AD in the population tested (which can be indexed as the prevalence of A+ and Tmod/high in that population), this is a fundamental point that needs to be detailed in both the abstract and the results, and will allow the reader to ascertain how representative the study sample is of the cohorts from which it was drawn, and any limitations in generalising to other settings where different prevalence may be seen. See specific recommendations below.

1. The abstract would benefit from clearly stating the prevalence of A+ and of THIGH+ at least, and qualifying "good discrimination" at the individual level (line 50)
   To keep the terminology consistent in the abstract, we have added: "Sixty % were A+, 36% Intermediate or Advanced stage and 9% Advanced."

   We have now revised the abstract Results section adding "Youden p217+tau thresholds gave sensitivity 0.77 [0.73 - 0.90], specificity 0.91 [0.80 - 0.95], PPV 0.84 [0.71 - 0.89] and NPV 0.88 [0.85 - 0.93] for combined Intermediate/Advanced stages. For Advanced stage alone sensitivity was 0.89 [0.79 - 0.97], specificity 0.82 [0.75 - 0.9], NPV 0.99 [0.98 - 1.0] but PPV only 0.33 [0.25 - 0.47]" and removed "good discrimination".

2. Introduction
   Line 67 – suggest clarifying "similar performance to p-tau217" to "similar performance to plasma p-tau217" as currently this line could be interpreted as p-tau217 measured in CSF.
   Thanks for this suggestion. It is now revised to: "It has shown similar performance to other plasma p-tau217 measures in detecting Aβ status in CSF".

3. Methods
   This paper would benefit from a flow chart showing how the final number of participants/samples was derived from the parent cohorts. Please provide the inclusion and exclusion criteria applied to arrive at the final sample number, as well as a reference, or further detail in the supplementary information, regarding the ADNeT cohort in particular. Overall from looking at the reference provided for AIBL it is apparent that individuals with MCI and AD dementia were recruited from memory

clinics but there was a mix of memory non-complainers and memory complainers among those classified as cognitively normal, and it would be helpful to know how they were recruited. It is interesting to note (from Results table 2) that those with A+THIGH+ were significantly younger than those in other groups and it would be helpful to know why that may have occurred at recruitment level (e.g. is there a weighting toward younger individuals with dementia being recruited rather than older individuals, and how that compares across the cohorts)?

We have added more information to the Methods, Participants section to address your questions:
"Since 2018, all participants recruited to the AIBL observational study and the allied ADNeT trial screening program have had ^{18}F-MK6240 tau PET, ^{18}F-NAV4694 Aβ PET and blood collection at entry. At the time of recruitment for this study, 506 participants in AIBL and ADNeT had both PET scans and blood assay results, and all were included in the current study. Details of AIBL recruitment and evaluation are described elsewhere and involved both advertising to the general community and referrals from memory disorder specialists[2]. ADNeT participants were evaluated as per AIBL methods and criteria. Briefly, participants were clinically classified as cognitively unimpaired (CU), mild cognitive impairment (MCI), Alzheimer's disease (AD) dementia or non-AD dementia, by a multi-disciplinary panel blind to PET imaging and blood assays results. Participants with diagnostic features of other neurodegenerative diseases were excluded. A diagnosis of CU required performance within 1.5 standard deviations (SD) of the published norms for their age group on selected neuropsychological assessments. A diagnosis of MCI or AD dementia followed internationally agreed clinical criteria[3, 4]."

The inclusion criteria are also reported in the "nr-reporting-summary.pdf" file, under "*Sample size*".

AIBL, as described in Ref 11*, is a very well-characterised study with hundreds of papers published based on AIBL data, since the AIBL study commencement.
Fowler C, et al.* Fifteen years of the australian imaging, biomarkers and lifestyle (AIBL) study: progress and observations from 2,359 older adults spanning the spectrum from cognitive normality to Alzheimer's disease. *J Alzheimers Dis Rep* **5**, 443-468 (2021).

The fact that A+THIGH+ are significantly younger might be that older people at Advanced stage of AD are less likely to volunteer for research or that indeed younger persons with dementia have more tau than older persons with dementia. It is a highly consistent finding across tau PET research sites that age has a positive association with tau-positivity in CU (irrespective of Aβ-status) but a negative relationship with tau-positivity in Aβ+ MCI and dementia (For an example, please see Figure 3 of preprint Rowe et al. 2022). We continue to recruit for tau PET and hopefully will eventually be able to answer this question.

4. It would be beneficial to include some detail on the actual testing of the samples eg singlicate vs duplicate testing, and how harmonization across testing carried out in 2 laboratories was ensured beyond stating that "assay scales measured from both laboratories were comparable" (line 141-142). Was a bridging study or at least sharing of common control samples employed across the two laboratories/two sets of

quantifications? Were there different reagent batches used even within each laboratory’s quantifications? These details are interesting to those in the field as we move toward testing the performance of these types of assay in the real world across laboratories and across reagent batches.
We have cited a paper by Triana-Baltzer et al. (2021) that reports the basic performance of the plasma p217+tau assay.
Triana-Baltzer et al. (2021) state:
“Here we describe a highly sensitive, quantitative, robust, and scalable method for measuring p217+tau in human plasma. The method has a dynamic range of 2 to 10,000 fg/mL and reports all plasma samples (including healthy controls) in this linear range. The method has good inter- and intra-test precision (< 20%) and dilution linearity in the range of quantifiable signal (2–6x).“ Page 11 of Triana-Baltzer et al. (2021) PDF.
“…, in Palmqvist et al, 25% of HC subjects and 7% of AD subjects had MSD p217tau plasma measurements below limit of detection, while here we show that all HC and AD subjects had Simoa p217+tau plasma measurements in linear range.” Page 13 of Triana-Baltzer et al. (2021) PDF.

All samples in our study were measured in duplicate in a given run. This is added to the Methods section: “The samples were measured in duplicate”.

Some samples were tested again in the 2nd batch (2nd cohort at the Quanterix), which allowed for the correlation assessment presented below (left panel). We consider this an adequate concordance between the two cohorts considering it is a) a different site/instrument/technician, b) several years later (i.e., sample age), and c) different lot of kit. Not only was the Spearman’s correlation quite high, but the absolute concentrations reported are also very similar. Hence, no normalization was needed. In addition, the distribution of concentrations seen across the entire set of cohort 1 and cohort 2 samples was not different, presented below (right panel).

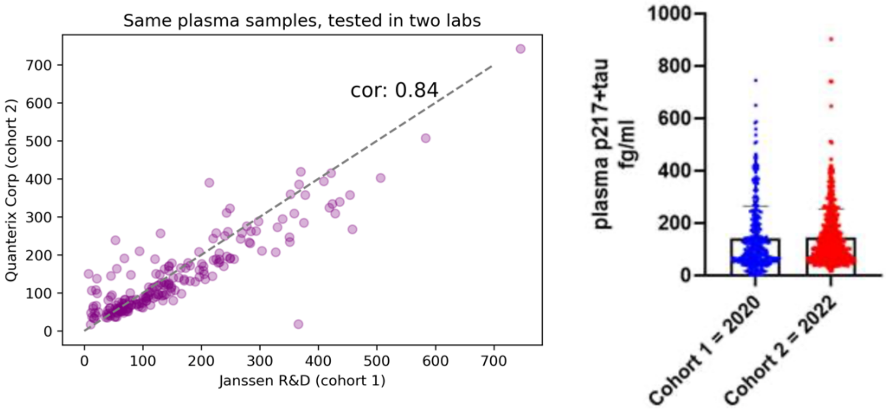

Additional information is added to the manuscript: “The correlation between the samples common to both cohorts was 0.84 (Spearman’s correlation). Assay scales measured from both laboratories were comparable and no normalization or transformation was required”.

5. Statistical methods
Looking at the dot plot overlaying the box plot on Figure 1, it is unlikely that the

distributions of plasma p217+tau within each AT group met the criteria for parametric testing with ANOVA and Tukey HSD (namely there is a skew more evident in some groups, and clear inhomogeneity of variances across the groups) so it is likely that a non-parametric method of comparison would be more appropriate (eg Kruskal-Wallis ANOVA followed by post hoc Dunn's test) and the more appropriate summary effect size would be median fold change rather than Cohen's D. I would suggest that these tests and values at least be included in supplementary information as an alternative.
Thanks for this suggestion. As per your recommendation, we have now revised the Statistical Methods section: "Between-group comparisons for quantitative variables were conducted using one-way analysis of variance (ANOVA) followed by Tukey's Honest Significant Difference (HSD) where residuals were normally distributed, and the Kruskal-Wallis test followed by Dunn's test for non-normally distributed residuals. Categorical (Gender, Apolipoprotein E (*APOE*) ε4 allele carriership, clinical stage) comparisons were performed with chi-square (χ^2) test. Values are reported as mean ± standard deviation (SD) when normally distributed and median ± interquartile range (IQR) when skewed. To investigate the magnitude of differences in plasma p217+tau concentration across the PET stages, effect sizes were estimated using Cohen's *d,* as well as median fold change in concentrations". Accordingly, Figure 1, Table 2, Supplementary Table 1, Supplementary Table 2, and Supplementary Table 3 are revised to reflect these changes.

6. Results
The results at group level are framed in terms of AUC and the sensitivity, specificity, PPV and NPV obtained using single cut-points at Youden's index. However an increasingly favoured approach for p-tau217 is to use two cutpoints that fix sensitivity and specificity (eg at 95% for both), and employ a grey zone – see Brum et al. 2023 (DOI: 10.1038/s43587-023-00471-5). It would be beneficial to include a similar exploration of the PPV and NPV obtained using this approach.
The main limitation of plasma p217+tau, based on our data, seemed to be in discriminating the A+T_{HIGH}+ stage without too much overlap with A+ T_{MOD}+. We agree that for this discrimination other thresholds could be explored.

We have added a Supplementary Figure 1, illustrating the effect of using three different thresholds: 90% sensitivity, 90% specificity and 95% specificity thresholds.

We have revised the Results section to reflect this addition :
"We also evaluated thresholds chosen for 90% sensitivity, 90% specificity and 95% specificity for A+T_{HIGH}+ (see Supplementary Figure 1) but no threshold was able to provide both satisfactory sensitivity and PPV (e.g. in CI a 90% sensitivity threshold gave PPV of only 32%; a 95% specificity threshold gave PPV of 70% with only 33% sensitivity). Thus adjusting the threshold to improve the PPV resulted in substantially reduced sensitivity due to the high degree of overlap with Intermediate stage AD, as can be seen in Figure 1."

Added supplementary Figure 1:

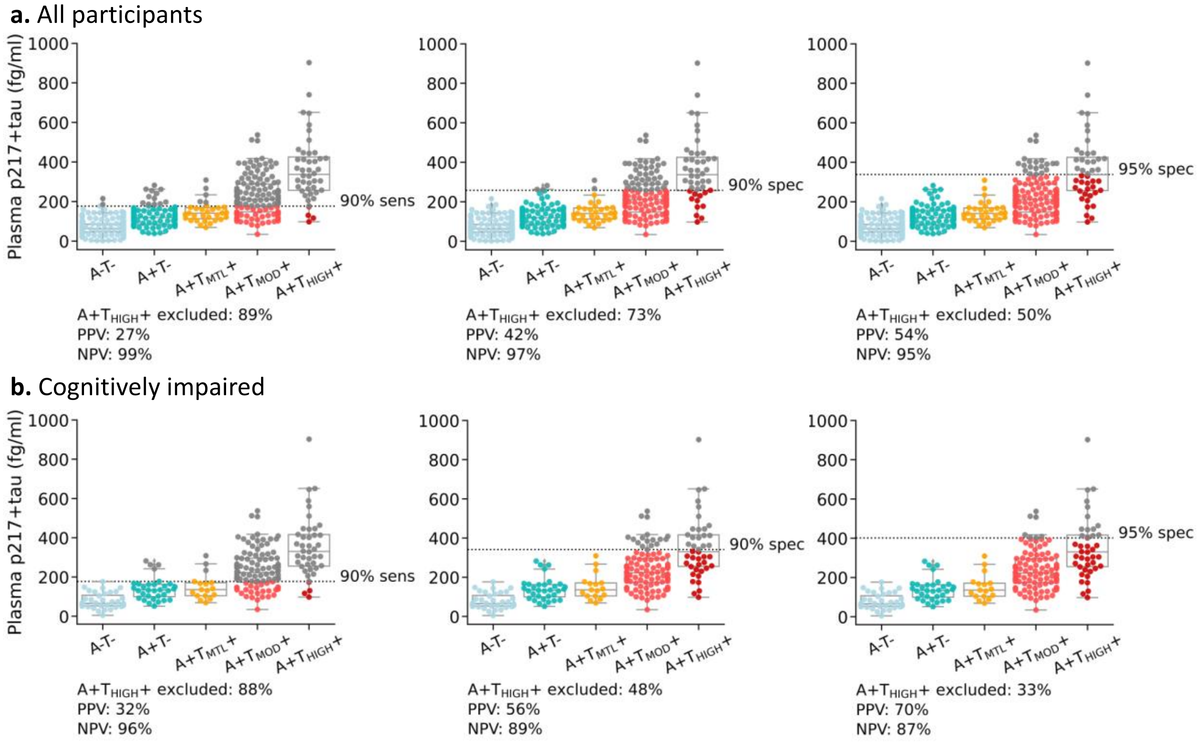

7. Lines 305-317 detail a sub-analysis within the group of individuals who have p217+tau >177.4 fg/ml; to determine the utility of a further cut-point based on ROC analysis within this sub-group, in discriminating those with THiGH+ from those in other disease stages. It would be helpful to know the size of this analysis group and the split of THIGH+ vs Tother within it, as if the T groups are significantly imbalanced in terms of numbers, AUC may overstate the discriminant value of this biomarker at this end of the spectrum of values.
The total number of participants in our sample with p217+tau > 177.4 fg/ml was 117. The breakdown of PET stages among these 117 was as follows:
    - A+T$_{HIGH}$+ = 37
    - A+T$_{MOD}$+ = 73
    - A+T$_{MTL}$+ = 3
    - A+T- = 4

We have added "(total n = 117, A+T$_{HIGH}$+ = 37)" to the Results section, under the sub-heading "*Two step discrimination between Intermediate and Advanced disease stages*".

8. While the individual level predictions of disease stage based on the logistic regressions (p217+tau as either continuous predictor or in 80 fg/ml bands) are interesting, they demonstrate the difficulty in predicting disease stage beyond the extremes of A-T- and A+THIGH+ based on this biomarker alone. While the authors state that this means that "One instead should consider an individual participant's likelihood for belonging to each of the stages based on their p217+tau level as illustrated by Figure 3.", it is difficult to see how a clinician would interpret for example a p217+tau value of 160 pg/ml in a cognitively impaired individual which according to figure 3B would confer equal probability of A+/Tnon/MTL+ and A+TMod+ (each around 40%) and equal lower probability of A-T- and A+THIGH+

(each around 5-10%). This should be recognized as a limitation in the use of individual level prediction that may or may not be improved by incorporation of other individual level factors (as the authors recommend) into risk prediction models.
We agree with these limitations and to more effectively reflect this, we have made major revisions to the paper.

Amongst the many changes we have made to clarify this issue are:

Abstract now includes:
"Youden p217+tau thresholds gave sensitivity 0.77 [0.73 - 0.90], specificity 0.91 [0.80 - 0.95], PPV 0.84 [0.71 - 0.89] and NPV 0.88 [0.85 - 0.93] for combined Intermediate/Advanced stages. For Advanced stage alone sensitivity was 0.89 [0.79 - 0.97], specificity 0.82 [0.75 - 0.9], NPV 0.99 [0.98 - 1.0] **but PPV only 0.33 [0.25 - 0.47].**
Conclusion: In addition to accurately screening for A+ individuals, plasma p217+tau may be useful to identify a combined Intermediate/Advanced stage AD cohort or pre-screen to reduce the tau PET required to identify Advanced stage AD individuals"

Results include the following under the "Discrimination between A+T_{HIGH}+ and combined A-T-/A+$T_{None/MTL}$/A+T_{MOD}+" sub-heading:
"In CI the Youden index provided a threshold of 230.3 fg/ml [205.4 - 299.5 fg/ml] giving sensitivity of 0.84 [0.69 - 0.95], and specificity 0.71 [0.64 - 0.88] (Supplementary Table 5). With PPV of 0.39 [0.31 - 0.58] and NPV of 0.95 [0.92 - 0.98], p217+tau effectively excluded Advanced stage AD when below the Youden threshold, but did not accurately predict Advanced stage when above, due to the inclusion of many Intermediate stage AD participants"

Also added to the results section:
"We also evaluated thresholds chosen for 90% sensitivity, 90% specificity and 95% specificity for A+T_{HIGH}+ (see Supplementary Figure 1) but no threshold was able to provide both satisfactory sensitivity and PPV (e.g. in CI a 90% sensitivity threshold gave PPV of only 32%; a 95% specificity threshold gave PPV of 70% with only 33% sensitivity). Thus adjusting the threshold to improve the PPV resulted in substantially reduced sensitivity due to the high degree of overlap with Intermediate stage AD, as can be seen in Figure 1"

In Discussion:
"…, the value of plasma p217+tau appears to lie in the detection of A+ individuals and to reduce the required number of tau PET for trial recruitment by defining a combined Intermediate/Advanced AD stage cohort where tau PET can then be used to separate Advanced from Intermediate stage. Similarly with a 95% NPV for Advanced stage AD when applied across all CI participants, plasma p217+tau may be used to screen out persons unlikely to have Advanced AD, with tau PET then required to separate Advanced AD from lower stages in those above the plasma p217+tau threshold"

"The overlap between the range of plasma p217+tau levels was again apparent in a risk prediction model using p217+tau as the predictor and estimated probability scores for an individuals' absolute risk of falling within a disease stage. With the overlap in

likelihood functions for most PET stages, confident staging of an individual with plasma p217+tau alone is limited to very low or very high levels (Figure 3)."

"To conclude, plasma p217+tau performs well for detecting persons with brain amyloid and discriminating those with Intermediate or Advanced stages from those at a lower stage, a useful property to reduce the number of tau PET required when screening for individuals at a particular stage of AD"
